# Feasibility of a tailored and virtually supported home exercise program for people with multiple myeloma using a novel eHealth application

**DOI:** 10.1177/20552076221129066

**Published:** 2022-10-11

**Authors:** Graeme M. Purdy, Chris P. Venner, Puneeta Tandon, Margaret L. McNeely

**Affiliations:** 1 Department of Physical Therapy, Faculty of Rehabilitation Medicine, 3158 University of Alberta, Edmonton, Alberta, Canada; 2 60294 Cross Cancer Institute, Edmonton, Canada; 3 Division of Gastroenterology, Department of Medicine, Faculty of Medicine and Dentistry, 3158 University of Alberta, Edmonton, Canada

**Keywords:** Myeloma, cancer, exercise, physical activity, tailored, eHealth

## Abstract

**Introduction:**

eHealth exercise interventions have the unique ability to leverage the benefits of in-person programming (tailoring and supervision) with the benefits of home programming (flexibility). There may be a role for eHealth-delivered exercise for people with multiple myeloma (MM), as exercise tailoring and supervision are critical for successful outcomes due to the significant impacts/risks of myeloma-related side effects. The purpose of this study was to determine the safety, feasibility, and preliminary efficacy of a 12-week virtually supported eHealth exercise program.

**Methods:**

Participants with MM completed a 12-week virtually supported home exercise program involving virtually supervised group workouts, independent workouts, and aerobic exercise. Tailoring was facilitated by the functionality of HEAL-Me, a novel eHealth app. Participants completed virtual fitness assessments and questionnaires at baseline and week 12.

**Results:**

Twenty-nine participants consented, 26 completed all follow-up testing (90%). Exercise adherence was 90% (group), 83% (independent), and 90% (aerobic). No serious adverse events (grade ≥3) occurred. Significant improvements were found for quality of life and physical fitness. There was a high level of program/app satisfaction: 96% of participants agreed or strongly agreed that the exercise program was beneficial, 93% found it enjoyable, 89% were satisfied or very satisfied with delivery through the HEAL-Me app, and 48% felt that the eHealth program helped them manage cancer-related symptoms and side-effects.

**Conclusion:**

An eHealth intervention that is individually tailored and includes virtual supervision and active support from the healthcare team is feasible and acceptable to people with MM. The findings from this study warrant investigation using a large-scale randomized controlled trial.

## Introduction

Multiple myeloma (MM) is a plasma cell cancer that accounts for roughly 1% of cancer diagnoses.^1^ MM causes osteolytic bone destruction,^2^ leading to pain, reduced mobility, fatigue, and increased fracture risk.^3^ Fortunately, treatment advances in recent decades have increased median survival from 3 to 6 years, meaning patients are living longer.^4^ However, patients are living with significant side effects from their treatment, including fatigue, myopathy, neuropathy, and pain.^5^ Care strategies are needed to improve patient function in the face of this lasting symptom burden.^6^

Exercise can improve the lived experience of people living with cancer.^7^ Guidelines show that exercise improves cancer-related health outcomes including fatigue, anxiety/depression, physical functioning, and quality of life.^7^ Unfortunately, these guidelines are based on studies in common cancers and may not apply to MM, given the unique disease- and treatment-related impairments. Specifically, myeloma bone disease leads to lytic lesions, which in turn can lead to fractures, pain, deformities, mobility issues, and neurological deficits.^8^ As such, myeloma bone disease poses a challenge for exercise delivery for two reasons: (1) exercise may be more likely to induce long bone fractures in this population compared to healthy individuals; (2) individuals may be hesitant to exercise due to a psychological fear of exercise triggering a fracture.^9^ In MM specifically, there is a paucity of exercise trials,^10^ with some studies showing promise in physical functioning,^11–14^ and others reporting non-significance.^15–19^ More research is required to determine what exercise volumes/types are appropriate for people with MM.

Tailoring and supervision may be key program characteristics to ensure successful exercise programming for people with MM,^20^ but flexibility is another important consideration.^21^ Home programs offer flexibility but lack supervision and support while in-person programs at hospitals or fitness centers can be logistically challenging for participants, leading to adherence and completion issues.^20^ eHealth programming could leverage the benefits of in-person programming (supervision and tailoring) and home programming (flexibility). Indeed, eHealth interventions are being increasingly researched in the field of exercise oncology.^22^ Recent trials have found eHealth interventions to be feasible and acceptable in older adults with cancer,^23,24^ individuals with thoracoabdominal malignancies,^25^ and individuals with metastatic prostate cancer.^26^ Other trials identify the promise of eHealth interventions for outcomes such as fatigue, strength, pain, and functional capacity in various cancer populations.^27,28^

The purpose of this study was to determine the safety, feasibility, and preliminary efficacy of a 12-week virtually supported home exercise program. The program progressed the activity of people with MM as recommended in the 2019 Exercise Guidelines for Cancer Survivors. We hypothesized that the 12-week program would prove safe and feasible for people with MM and would demonstrate preliminary efficacy with key physical function and quality of life outcomes achieving minimally important differences.

## Methods

### Study design

The Myeloma Progressive Resistance and Aerobic Exercise Study (MY PROGRESS) was a single group pre-post feasibility study. The study received ethics approval from the Health Research Ethics Board of Alberta: Cancer Committee on 26 August 2020 (HREBA.CC-20-0201) and was registered at www.clinicaltrials.gov (NCT04484714). Participants provided informed written consent prior to enrollment. This article follows the CONSORT Statement extension for randomized pilot and feasibility trials.^29^ Although this study is not randomized, many of the CONSORT principles from this extension still apply.^29,30^ The target sample size was 25.^31^

### Participants

Using convenience sampling, participants meeting the following criteria were recruited: ≥18 years old; MM diagnosis; in one of three categories: (i) transplant ineligible, first-line treatment, (ii) transplant eligible, >3 months post-transplantation, and (iii) relapsed/recurrent myeloma with ≥1 prior line of treatment; ability to provide informed written consent in English. Recruitment occurred through (i) outpatient appointments at local cancer centers (oncology staff provided information to eligible patients), (ii) study presentations for the local myeloma patient support society, and (iii) eligible former participants of the Alberta Cancer Exercise program (ACE).^32^

Participants were screened using the Physical Activity Readiness Questionnaire for Everyone (PAR-Q+)^33^ and a cancer-specific intake form (which provided details about treatment history, bone involvement, as well as ongoing side effects, and issues the participant was experiencing, including, fatigue, pain, neuropathy, osteoporosis or bone loss, muscle or joint issues, etc.) by an exercise physiologist and approved by their physician prior to enrollment. Baseline physical activity level was determined using the Godin-Shephard Leisure-Time Physical Activity Questionnaire.^34^ Bone disease was considered but was not exclusionary, to capture a sample reflective of the real-world MM population. Physician approval was contingent on the stability of symptoms and reasonable optimization of pain control. At approval, the physician shared the participant's history and/or location of bony lesions with the exercise team. Exclusion criteria included: physician-determined inability to exercise safely at home based on clinical judgment; diagnosis of amyloid light-chain amyloidosis, solitary plasmacytoma, or Waldenstrom macroglobulinemia; too frail to partake in home programming (i.e., cannot perform 1 sit-to-stand or balance for >3 s on one foot).

### Procedures

Programming was delivered by a kinesiologist with >3 years of exercise oncology experience with oversight from an exercise physiologist and physiotherapist. Participants received a 12-week tailored exercise program based on the 2019 Exercise Guidelines for Cancer Survivors.^7^ The goal was to progress participants to moderate-intensity resistance training ≥2 times/week and moderate-intensity aerobic exercise ≥90 min/week by study completion.

**Figure 1.**
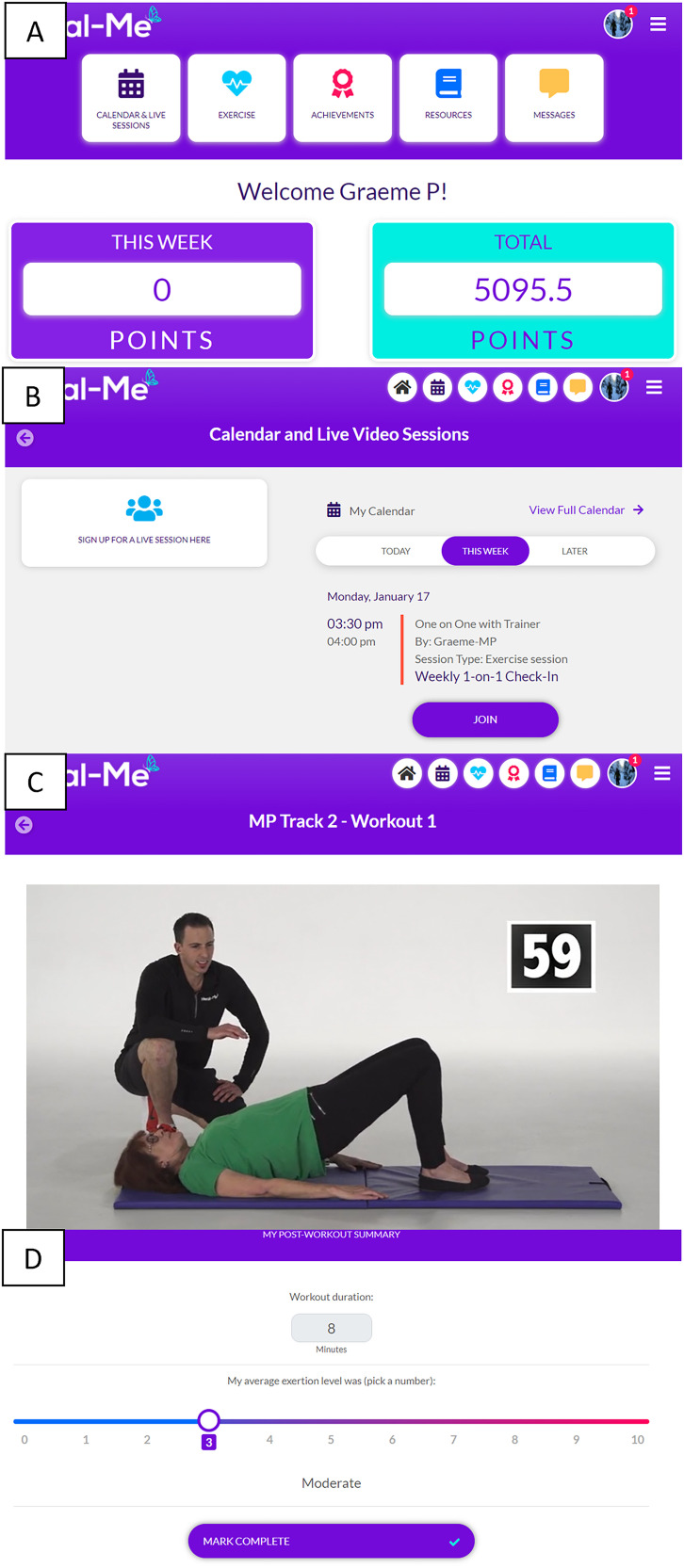
Screenshots from the HEAL-Me app showing the (A) home page of the app; (B) calendar and live session section, where participants connect to virtually supervised group workouts and check-ins with their trainer, (C) participant view of an independent home workout, and (D) workout logging page.

The program was delivered through HEAL-Me, an e-Health technology.^35^ HEAL-Me is an online non-commercial application (app) developed by a University of Alberta research team led by a MY PROGRESS investigator (PT). HEAL-Me offers flexible, tailored home exercise programming using: (i) virtually supervised group workouts; (ii) independent home workouts; and (iii) independent aerobic exercise. See Figure 1 for images of the main sections in the app and Section 2 of the Online Supplement for additional app information.

*Resistance exercise.* The resistance exercise component involved 60-min virtually supervised group workouts as well as assigned independent workouts on the app. Participants progressed in exercise frequency and intensity over the 12-week program (Table 1). Workouts lasted 60 min (10-min aerobic-based warm-up, two rounds of an 8-exercise circuit with a work-to-rest ratio of 60 s:30 s, 5-min cool-down stretch). Circuits involved two cardio, two upper body, two lower body, one balance, and one core exercise. Muscle groups specifically impacted by MM were preferentially worked over others, including proximal limb muscle groups,^36,37^ the muscles of the core, and the back. As recommended by the International Bone Metastases Exercise Working Group, an overarching emphasis was placed on exercise performance to ensure proper technique, postural alignment, and tempo (controlled movement). Specifically, principles of exercise prescription included avoidance of exercises with high fall risk, extremes of spinal movements (flexion, extension, and rotation), and rapid and/or weight-loaded end-range movements that could potentially impact the location of lytic lesions.^38^ Each workout had a secondary circuit that followed the initial one, which alternated between (1) a core exercise sequence: two sets of two core exercises, (2) an 8-min balance sequence, and (3) additional stretching. Independent workouts were assembled from a bank of >150 exercises on the HEAL-Me app. At the program start, participants were matched to one of four program start-points based on their fitness (Table 1). From there, participants followed the program's pre-set myeloma-specific routine progression, with a slightly new routine prescribed each week. Adaptations were made to tailor each week's routine to the participant to ensure exercise safety, quality performance, and enjoyment. Group classes offered 2 to 3 levels of difficulty per exercise, and participants were matched to the option that was most appropriate for them. Additional resistance exercise program details are available in Table 1. An overview of both the lesson plans used for virtually supervised group workouts and the independent workout templates are available in Sections 3 and 4 of the Online Supplement, respectively.

**Table 1.** Intervention description using the template for description and replication (TIDieR).

MY PROGRESS		
Materials and procedures	Participants received initial one-on-one app training followed by access to the Healthy Eating, Active Living, and Mindful Energy (HEAL-Me) application for 12 weeks. Participants were also given exercise equipment (i.e., dumbbell weights and exercise bands) if they did not have sufficient equipment at home prior to the intervention. Participants were able to use whatever additional exercise equipment they had access to at home (e.g., treadmill, bike, elliptical, and exercise ball).	
Providers	Personnel structure aligned with recommendations from the International Bone Metastases Exercise Working Group. ▪ Programming delivery: kinesiologist with >3 years of exercise oncology experience ▪ Exercise screening: cancer-specific exercise physiologist ▪ Program oversight: physiotherapist with >20 years of experience in cancer rehabilitation ▪ Medical approval: hematologist/oncologist	
	**Resistance exercise**	**Aerobic exercise**
Guiding principle(s)	Postural alignment, controlled movement, proper technique	Progressive overload of the cardiovascular system
Location	Participant's home (supervised and independent)	Home or outdoors (independent)
Program start point	▪ Considerations: baseline physical assessment results (2-min step test, 30-s sit-to-stand, plank duration, timed single leg balance test), previous experience with resistance exercise, bone disease and comorbidities, and participant comfort level ▪ Initial resistance training program selected based on a clinical judgment from the study kinesiologist using the above considerations, in consultation with the rest of the multidisciplinary research team ▪ Rough criteria corresponding to the assignment of a starting point (note: exceptions were made based on clinical judgment): ▪ Starting Point #1: ≤50 reps in 2-min step test; ≤7 reps in 30-s sit-to-stand; ≤20 s in single leg balance test; inability to complete plank test due to comfort and/or bone disease ▪ Starting Point #2: 50–75 reps in 2-min step test; 7–12 reps in 30-s sit-to-stand; 10–30 s in single leg balance test; plank duration ≤50 s ▪ Starting Point #3: ≥75 reps in 2-min step test; ≥12 reps in 30-s sit-to-stand; ≥20 s in single leg balance test; plank duration ≥50 s ▪ Starting Point #4: ≥85 reps in 2-min step test; ≥15 reps in 30-s sit-to-stand; ≥40 s in single leg balance test; plank duration ≥100 s	▪ Criteria: baseline physical activity level (Godin Questionnaire) and 2-min step test score ▪ Progression 1: baseline aerobic exercise <90 min/week → progression to 90 min/week of moderate-to-vigorous exercise by week 12 ▪ Progression 2: baseline aerobic exercise ∼90 min/week → maintain 90 min/week of moderate-to-vigorous exercise OR progress to 150 min/week (if interested)
Frequency	▪ Virtually supervised group workout: 1 time/week ▪ Independent workout: 1 time/week for weeks 1–6, 2 times/week for weeks 7–12	▪ Volume-based exercise prescription ▪ Target frequency of 2–5 times/week, as the target volume ↑
Intensity	Target intensity set based on Borg's 0–10 scale for RPE. ▪ Weeks 1–3: RPE 3 ▪ Weeks 4–9: RPE 4 ▪ Weeks 10–12: RPE 5	Target intensity set based on Borg's 0–10 scale for RPE. Monitored using the talk test ▪ Weeks 1–3: RPE 3 ▪ Weeks 4–9: RPE 3/4 ▪ Weeks 10–12: RPE 4
Workout structure	Total time: 60 min Warm-up: Option 1: 10-min light-to-moderate aerobic exercise on treadmill, elliptical, bicycle Option 2: 10-min light-to-moderate-intensity aerobic dance exercise video on the HEAL-Me app Main circuit (2 rounds, 8-exercise sequence, work-to-rest ratio: 60 s work, 30 s rest): 2 cardio, 2 upper body, 2 lower body, 1 balance, and 1 core Secondary circuit (alternated each workout): • Core sequence: 2 sets of two core exercises, 60 s work, 30 s rest • Balance and grip stretch sequence: 8-min balance sequence (dynamic and static balance using a narrow base of support ± unipedal stance, with grip strength work using towel or ball) • Stretching sequence: 5 additional minutes of stretching prior to cool-down Cool-down: 5 min of light stretching	Modality of choice based on participant preference and access to equipment (e.g., walking outdoors, use of a treadmill, elliptical, stationary bike, cardio dance, etc.). Warm-up: 5 min of light-to-moderate aerobic exercise (if desired/preferred) Main session: aerobic exercise at target RPE Cool-down: 5 min of light aerobic exercise and/or light stretching (encouraged)
Safety	▪ Avoid rapid and/or weight-loaded end-range movements impacting the location of lytic lesions ▪ Avoid high-impact activities for participants at risk of spine fracture/history of spine fracture ▪ Avoid activities with excessive spinal rotation/twisting ▪ Avoid activities that hyperflex/extend the trunk ▪ Avoid activities that put participants at excessive risk of falling ▪ Avoid activities that put excessive load on a bone lesion site (consider lever length and positioning) ▪ Safe workout environment—space free from tripping hazards and use of proper footwear ▪ Overarching emphasis on proper technique, postural alignment, and controlled tempo of exercise Monitoring: ▪ Before exercise: regular one-on-one “check-ins” (see below) were conducted to monitor participant health and progress and to implement program adaptations as needed ▪ During group workouts: virtual supervision—cameras turned on and participants in view of camera during all group workout classes ▪ During independent workouts: participants monitored for symptoms (e.g., pain, dizziness, shortness of breath, etc.) and were instructed to notify the study kinesiologist if symptoms arose/persisted ▪ During aerobic exercise: intensity monitoring using talk test participants monitored for symptoms and were instructed to notify the study kinesiologist if symptoms arose/persisted ▪ Following exercise: participants recorded their exertion level in the HEAL-Me app. Participants were instructed to monitor for and notify the study kinesiologist if any symptoms arose/persisted after exercise.	
Tailoring and modifications	Virtual one-on-one “check-ins” were conducted during weeks 1–3, 5, 7, 9, and 11 to facilitate tailoring and education through discussions around program design, adaptations, technique, and exploration of challenges and successes of participants. Objectives of adaptations: match the prescribed exercises with the participant's abilities, goals, and preferences with a primary goal of ensuring exercise safety and quality performance and a secondary goal of ensuring exercise enjoyment. High-level principles of progressions: ▪ Cardio: seated or supported exercise → free-standing exercise → increased speed ± additional movement (e.g., seated march → standing march → step-ups) ▪ Lower body: isolation movement or movement with support → compound movement → compound ± resistance or impact (e.g., step back with chair support → reverse lunge → forward lunge or sit-to-stand → bodyweight squat → weighted squat with knee raise) ▪ Upper body: seated, below-shoulder exercises → increased range of motion and/or resistance in seated, standing, or supine position → addition of lower body challenge (e.g., seated row with a band → standing bow-and-arrow band pull → bow-and-arrow band pull in 1/4 squat) ▪ Core: basic core contraction with minimal impact on spine → increased difficulty by the use of gravity, body position, or reach, resistance → further increased difficulty (e.g., wall plank → knee plank → full plank or legs-only bird dog → bridge → bridge with band) ▪ Balance: decrease base of support → single leg postures → add movement (e.g., tandem stance → single leg stance → single leg alphabet) Indications for progression: ▪ Completion of resistance exercise for ≥2 weeks with proper form/technique ▪ Minimal/usual/expected level of fatigue and muscle soreness/discomfort ▪ No increased pain after exercise ▪ ±exercise is described as not providing enough challenge/stimulus to a participant Indications for regression: ▪ Excessive fatigue after exercise ▪ Muscle soreness lasting >48 h or increased pain after exercise ▪ Inability to perform an exercise with proper/safe form or technique, despite appropriate coaching, cueing, and/or education	
Fidelity	▪ Monitored by study kinesiologist ▪ Methods of monitoring adherence and tailoring: adherence tracking through the HEAL-Me eHealth app tracking software, virtual one-on-one check-ins, and recording of adaptations to individual programs and the reasons for those adaptations ▪ Methods for monitoring safety: monitoring of symptoms and recording of minor and serious adverse events	

MY PROGRESS: Myeloma Progressive Resistance and Aerobic Exercise Study; RPE: rating of perceived exertion.

*Aerobic exercise.* The aerobic exercise component progressed participants from their current baseline level of aerobic exercise up to ≥90 min/week of moderate-intensity aerobic exercise (Table 1). Participants selected their preferred aerobic exercise (e.g., walking, elliptical, or cycling) and completed sessions independently. Participants who were exercising below aerobic exercise recommendations gradually progressed up to 90 min of moderate-intensity aerobic exercise per week based on American College of Sports Medicine progression principles.^39^ Participants monitored their intensity using the talk test to confirm they were exercising below anaerobic threshold by ensuring they were “just capable of talking” (i.e., breathing harder but still capable of talking without gasps of air between words) while exercising^40^ and recorded their aerobic exercise using the app's activity tracking portal, which includes a box for recording duration and a slider scale for rating of perceived exertion. Additional aerobic exercise program details are available in Table 1.

 Adherence was tracked directly in HEAL-Me. Reasons for missed sessions, adaptations to individual programs, and reasons for adaptations were recorded. Virtual one-on-one sessions were conducted on a regular basis (weeks 1–3, 5, 7, 9, and 11) to discuss program design, adaptations, and techniques, and to explore the challenges and successes of participants.

### Outcome measures

The primary outcome of this study was the protocol's feasibility. Feasibility was determined by uptake (≥21 participants consenting to the study over a 7-month recruitment period), completion (≥80% of consenting participants completing the 12-week assessment), safety (absence of serious adverse events related to the intervention, specifically events requiring reporting as per the local research ethics board standards—including exercise-related events that were life-threatening or required hospitalization), and adherence (completion of ≥75% of the exercise prescription). Adverse events were tracked and recorded as per the Common Terminology Criteria for Adverse Events (CTCAE version 5.0).

Questionnaires and virtual physical assessments were completed pre- and post-intervention (12 weeks) to evaluate secondary outcomes. Questionnaires were administered online using REDCap electronic data capture tools hosted and supported by the Women and Children's Health Research Institute at the University of Alberta.^41^ Physical assessments were completed virtually using the latest version of Zoom Meetings (Zoom Video Communications, San Jose, CA). The study's kinesiologist completed baseline assessments, and an independent kinesiologist completed post-intervention assessments. Physical assessments were used to determine aerobic exercise capacity (2-min step test),^42^ lower body muscle strength (30-s sit-to-stand test),^43^ core endurance (plank endurance test), and balance (one-legged stance).^44^ Upper body flexibility was assessed by active shoulder flexion range of motion using a goniometer.^45^ Lower body flexibility was assessed using the modified sit-and-reach test.^46^ Height and weight were abstracted from medical records. Questionnaires assessed the quality of life (Functional Assessment of Cancer Therapy (FACT)-MM),^47^ fatigue (Functional Assessment of Chronic Illness Therapy (FACIT)-Fatigue),^48, 49^ and symptom burden (Edmonton Symptom Assessment Scale (ESAS)).^50, 51^ Bone pain was assessed using the FACT-bone pain (BP).^52^ Neuropathy was assessed using the FACT/Gynecologic Oncology Group-Neurotoxicity 4 (GOG-NTX4).^53^ The physical, social, emotional, and functional subscales of the FACT are also reported. Finally, physical symptom burden and psychological symptom burden were assessed using the subscales from the ESAS.^50, 51^

### Statistical analysis

Demographics and feasibility measures are presented using descriptive statistics (mean ± SD or median (range) for continuous variables, frequency (percentage) for nominal variables). The normality of secondary outcomes was tested using the Skewness–Kurtosis test in Stata/MP. Data were analyzed using 95% confidence intervals (CIs) if normally distributed. Data were analyzed using asymmetric CIs and are presented as median along with the 25th and 75th quartiles and 5th and 95th percentiles if non-normally distributed. Analyses were conducted using Stata/MP 13.0 (StataCorp LLC, College Station, TX).

## Results

### Participants and feasibility outcomes

Recruitment occurred over 7 months: September 2020 to March 2021. Thirty-one participants initially expressed interest in the study and were screened for eligibility. One participant decided not to participate after discussing the study with the research team because they were starting a new clinical trial drug. A second participant had stage 4 colon cancer and was no longer being followed for MM, so was referred to a different exercise program delivered by our research lab that was more appropriate. As such, 29 participants consented (uptake: 4.1 participants/month). Consenting participants were recruited by oncology staff referral (*n* = 8), myeloma support society (*n* = 15), former ACE participants (*n* = 3), and Myeloma Canada (*n* = 3). Twenty-eight participants completed baseline testing and began the 12-week program (one participant did not begin the program due to bone pain that required medical intervention). The mean age of participants was 65 ± 8.4 years (50% males, Table 2). Four participants (14%) were transplant ineligible in first-line treatment, 8 participants (29%) were in first-line treatment post stem cell transplantation, and 16 participants (57%) had relapsed/recurrent myeloma and were in a second or later line of treatment. Given the small sample size, secondary outcome results were not stratified. Participants were a median of 35 months post-diagnosis (range: 9–164 months). Twelve participants (43%) were on maintenance therapy. Table 2 provides detailed participant characteristics. Of the 28 that started the program, 26 participants completed the program and follow-up fitness testing (92.9%), and 27 completed the follow-up questionnaires (96.4%). One participant passed away, the other experienced a spinal fracture unrelated to the exercise program so completed the 12-week questionnaires but not the fitness assessment. Six participants were unable to complete the sit-and-reach test (no family member at home to assist with a test (*n* = 5), apprehension to spinal flexion (*n* = 1)) and 6 participants were unable to complete the plank test (history of spinal injury or back pain (*n* = 5), inability to get onto the floor (*n* = 1)).

**Table 2.** Participant characteristics and demographics.

Current employment status	Frequency (%)
Age (years)	65.0 ± 8.4
BMI (kg/m^2^)	26.5 ± 4.9
Time since diagnosis (months)	35 (9–164)
No. of lines of treatment at the program start	2 (1–5)
**Sex**	Frequency (%)
Male	14 (50%)
Female	14 (50%)
**Marital status**	Frequency (%)
Never married	2 (7%)
Married	24 (86%)
Divorced	2 (7%)
**Education**	Frequency (%)
Completed high school	6 (21%)
Some university/college	4 (14%)
Completed university/college	15 (54%)
Some graduate school	1 (4%)
Completed graduate school	2 (7%)
**Family Income**	Frequency (%)
Did not disclose	6 (21%)
$20,000–59,999	6 (21%)
$60,000–99,999	9 (32%)
>$100,000	7 (25%)
**Current employment status**	Frequency (%)
Disability	8 (29%)
Retired	15 (54%)
Part-time	1 (4%)
Full-time	3 (11%)
Homemaker	1 (4%)
**Ethnic origin**	
Caucasian (White)	26 (93%)
Southern Asian	1 (4%)
Unknown (adopted)	1 (4%)
**Smoking status**	
Never smoked	9 (32%)
Ex-smoker	17 (61%)
Regular smoker	2 (7%)
**Drinking status**	
Never drank	1 (4%)
Ex-drinker	3 (11%)
Occasional or social drinker	16 (57%)
Social drinker	7 (25%)
Regular drinker	1 (4%)
**Current treatment**	
Lenalidomide	6 (21%)
Lenalidomide + dexamethasone	3 (11%)
Lenalidomide + daratumumab	3 (11%)
Lenalidomide + ixazomib	3 (11%)
Lenalidomide + daratumumab + dexamethasone	2 (7%)
Lenalidomide + ixazomib + dexamethasone	1 (4%)
Carfilzomib + dexamethasone	2 (7%)
Carfilzomib + cyclophosphamide + dexamethasone	1 (4%)
Ixazomib	1 (4%)
Bortezomib	1 (4%)
Bortezomib + CC92480 + dexamethasone	1 (4%)
Pomalidomide + cyclophosphamide + dexamethasone	1 (4%)
Pomalidomide + dexamethasone	1 (4%)
Pomalidomide + daratumumab + dexamethasone	1 (4%)
Surveillance	1 (4%)
**SCT information**	
One previous SCT	19 (68%)
>1 previous SCT	2 (7%)
SCT in the current line of treatment	11 (39%)
SCT in the previous line of treatment	10 (36%)
**Other disease information**	
Bone disease	24 (86%)
Previous radiation therapy	13 (46%)
Previous surgery^1^	8 (29%)
**Location of bone disease**	
Thoracic spine/ribs	16 (57%)
Lumbar spine	9 (32%)
Pelvis	9 (32%)
Femur	9 (32%)
Skull	7 (25%)
Humerus	4 (14%)
Cervical spine	2 (7%)
Other	3 (11%)
**Extent of bone disease**	
Minor (1 location)	6 (21%)
Moderate (2 locations)	9 (32%)
Major (≥3 locations)	9 (32%)
**Baseline physical activity**	
≥90 min of aerobic MVPA	8 (29%)
>0, <90 min of aerobic MVPA	4 (14%)
0 min of aerobic MVPA	16 (57%)
≥2 times/week of resistance MVPA	6 (21%)
1 time/week of resistance MVPA	2 (7%)
0 time/week of resistance MVPA	20 (71%)

BMI: body mass index; SCT: stem cell transplantation; MVPA: moderate-to-vigorous physical activity.

^1^ Includes vertebroplasty, kyphoplasty, or orthopedic surgeries related to myeloma.

Adverse events related (*n* = 4) and unrelated (*n* = 3) to the intervention were identified. Two cases of mild back pain (grade 1) occurred, one during a seated version of bridging (lumbar pain) and one during a low-impact jumping jack exercise (thoracic pain). Both cases were resolved within a few days and did not impact adherence. One case of moderate back pain (grade 2) occurred following a bridging exercise, resulting in a 6-day pause from exercise. One case of moderate-to-severe back pain occurred following a hip flexor stretch, resulting in a 7-day pause. This event fell into the category of grade 2 to 3 pain acutely but did not limit the participant's self-care activities of daily living in the days following, was not life-threatening, and did not require hospitalization. Thus, the event did not constitute a serious adverse event, given it did not meet full CTCAE criteria for a grade 3 back pain event.

One case of moderate-to-severe spinal fracture (grade 2 to 3) unrelated to the exercise intervention occurred when a participant had a fall in their bathroom. The participant discontinued exercise for the remainder of the program (2 weeks). One case of moderate back pain (grade 2) unrelated to the intervention occurred when a participant slipped on ice outdoors, resulting in a 4-day pause. Finally, one case of grade 2 arrhythmia unrelated to the exercise intervention occurred in a participant with a history of previous cardiac intervention, resulting in a 10-day pause while undergoing cardiology testing/clearance.

**Figure 2.**
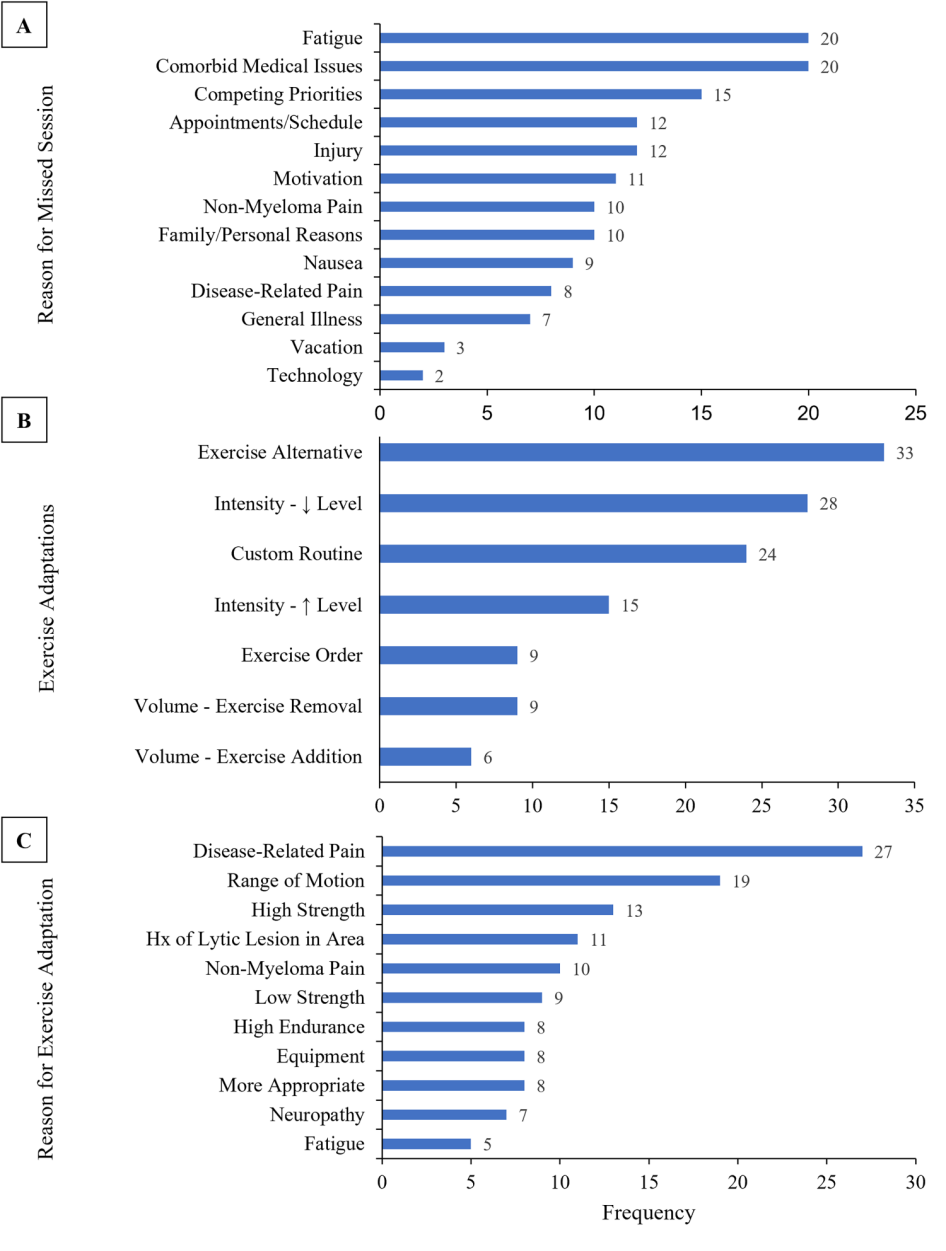
(A) participant-reported reasons for non-completion of exercise sessions (group workout, independent workout, and independent aerobic exercise) during the 12-week exercise program by frequency of occurrence. (B) Exercise adaptations made for participants during the 12-week exercise program by frequency of occurrence. Exercise alternative: replacement of one exercise with another one of similar difficulty and goal. Intensity—↓ level: an exercise of lower intensity, targeting similar muscle groups. Custom routine: all exercises were changed from the original template routine. Intensity—↑ level: an exercise of higher intensity than the original, targeting similar muscle groups. Exercise order: the order of the exercises in the routine was adjusted to make it easier for the participant to transition between exercises. Volume—exercise removal: one or more exercises were removed and not replaced. Volume—exercise addition: one or more exercises were added without removing an exercise. (C) Reason for individual exercise adaptations made during the 12-week exercise program by frequency of occurrence.

Participants completed 82.9% of independent home workouts, 89.9% of group workouts, and 89.7% of aerobic exercise. For aerobic exercise, 16 participants progressed from 40 min/week to 90 min/week, 5 participants maintained 90 min/week for 12 weeks, 3 participants progressed from 90 min/week to 150 min/week, and 4 participants maintained 150 min/week for 12 weeks. Reasons for missed exercise included: fatigue (*n* = 20), comorbid medical issues (*n* = 20), and competing priorities (*n* = 15) (Figure 2A). Exercise adaptations included: exercise alternatives (*n* = 33), decreased level of difficulty (*n* = 28), and custom routine (*n* = 24) (Figure 2B). Exercise adaptation reasoning included: disease-related pain (*n* = 27), limited range of motion (*n* = 19), and history of fracture/lytic lesion in the area (*n* = 10) (Figure 2C). A complete breakdown of all exercise adaptations made during the study is available in Section 1 of the Online Supplement (Supplemental Tables S1–S4).

### Satisfaction survey

There was a high level of program satisfaction (Table 3). Most participants agreed or strongly agreed that the exercise program was beneficial (96.3%, *n* = 26) and enjoyable (92.6%, *n* = 25) and felt the service provided by the program staff was excellent (92.6%, *n* = 25). Participants were satisfied with the HEAL-Me app, disagreeing or strongly disagreeing that the app was burdensome (88.9%, *n* = 24). While 48% of participants (*n* = 13) felt that the program helped them manage cancer-related symptoms and side effects, 48% of participants felt neutral about the program's benefits for symptoms and side effects.

**Table 3.** Participant satisfaction with the exercise program, program staff, and mobile app, based on a program satisfaction survey completed following the 12-week exercise program.

	Strongly disagree *n* (%)	Disagree *n* (%)	Neutral *n* (%)	Agree *n* (%)	Strongly agree *n* (%)
**Exercise program**					
The program was beneficial to me.	0 (0)	0 (0)	1 (3.7)	12 (44.4)	14 (51.9)
The program was enjoyable to me.	0 (0)	0 (0)	2 (7.4)	11 (40.7)	14 (51.9)
Completing the program helped me meet my health and wellness goals.	0 (0)	0 (0)	2 (7.4)	13 (48.1)	12 (44.4)
The program helped increase my knowledge related to the benefits of exercise for multiple myeloma	0 (0)	2 (7.4)	1 (3.7)	11 (40.7)	13 (48.1)
The program helped me manage symptoms and side effects related to my cancer and/or treatments	0 (0)	1 (3.7)	13 (48.1)	6 (22.2)	7 (25.9)
I would recommend the exercise program to others	0 (0)	0 (0)	0 (0)	4 (14.8)	23 (85.2)
**Program staff**					
The program staff made me feel comfortable	0 (0)	0 (0)	0 (0)	4 (14.8)	23 (85.2)
The program staff were knowledgeable	0 (0)	0 (0)	0 (0)	2 (7.4)	25 (92.6)
The program staff were supportive	0 (0)	0 (0)	0 (0)	3 (11.1)	24 (88.9)
The program staff worked with me to ensure the exercises were appropriate for my level of fitness and my symptoms	0 (0)	0 (0)	0 (0)	4 (14.8)	23 (85.2)
Overall, the service you received from the program staff was excellent	0 (0)	0 (0)	0 (0)	2 (7.4)	25 (92.6)
**Mobile app**					
It was a burden learning how to use the HEAL-Me App	17 (63)	5 (18.5)	5 (18.5)	0 (0)	0 (0)
It was a burden using the HEAL-Me App to exercise	19 (70.4)	5 (18.5)	3 (11.1)	0 (0)	0 (0)

### Secondary outcomes

A summary of secondary outcome results is available in Table 4. The mean difference in pre- versus post-intervention aerobic exercise capacity (2-min step test score) was +12.7 m (95% CI: 8.7–16.8). The mean difference in pre- versus post-intervention lower body muscle strength (30-s sit-to-stand score) was + 2.7 repetitions (95% CI: 1.6–3.8). The mean difference in pre- versus post-intervention core endurance (plank duration) was + 41.6 s (95% CI: 22.3–60.8 s). The mean difference in pre- versus post-intervention balance (timed single leg stance test) was + 8.7 s (95% CI: 4.6–12.8).

**Table 4.** Summary of secondary outcomes from pre-/post-intervention questionnaires and physical assessments.

Outcome	*n* (BL/12WK)	Baseline	12-Week	Mean change [95% CI]	Effect size	MID
**Physical assessments**						
2-min step test (steps)	28/26	68.6 ± 17.7	81.3 ± 16.0	12.7 [8.7 to 16.8]	1.28	NA
30-s sit-to-stand (repetitions)	28/26	13.1 ± 4.5	15.8 ± 4.3	2.7 [1.6 to 3.8]	1.00	> 2^60, 61^
Plank duration (s)	23/21	78.3 ± 46.0	119.9 ± 73.4	41.6 [22.3 to 60.8]	0.98	NA
Timed single leg stance test (s)	28/26	23.1 ± 13.3	31.8 ± 12.6	8.7 [4.6 to 12.8]	0.86	24^73^
Active shoulder flexion (degrees)	28/26	146.8 ± 11.7	149.4 ± 11.5	2.6 [0.7 to 4.5]	0.56	> 10^74^
Modified sit-and-reach (cm)	23/21	−5.8 ± 14.0	−2.2 ± 13.6	3.6 [1.6 to 5.7]	0.81	NA
**Questionnaires — normal distribution**						
FACT-MM (score)	28/27	111 ± 23	118 ± 19	7.3 [0.6 to 14.0]	0.43	NA
FACT physical (score)	28/27	20.3 ± 4.5	20.9 ± 4.0	0.6 [−1.0 to 2.2]	0.16	2–3^69^
FACT social (score)	28/27	21.8 ± 4.7	22.3 ± 4.9	0.5 [−0.7 to 1.7]	0.15	2–3^75^
FACT functional (score)	28/27	17.2 ± 4.3	17.9 ± 4.2	0.7 [−0.6 to 2.0]	0.21	2–3^69^
FACT emotional (score)	28/27	16.4 ± 4.9	18.5 ± 3.1	2.1 [0.6 to 3.6]	0.57	2–3^69^
FACIT-Fatigue (score)	28/27	35 ± 10	37 ± 8	1.7 [−1.4 to 4.8]	0.21	3^71^
FACT/GOG-NTX4 (score)	28/27	6.1 ± 3.5	6.9 ± 4.1	0.6 [−0.6 to 2.0]	0.21	NA
**Questionnaires — non-normal distribution**		Median (range)	Median (range)	Median [5th, 25th, 75th, 95th percentiles]		
FACT-BP (score)	28/27	49 (19–59)	49 (28−59)	0 [−11, −5, 6, 12]	0.00	NA
ESAS global (score)	28/27	10.5 (0−43)	12 (0−35)	1 [−16, −9, 11, 17]	−0.04	3^76^
ESAS physical (score)	28/27	6 (0−27)	2 (0−12)	−4 [−12, −8, −2, 0]	0.85	2^76^
ESAS psychological (score)	28/27	2 (0−15)	8 (0−25)	4 [−6, 0, 10,17]	−0.53	3^76^

BL/12WK: baseline/12-week; MID: minimally important difference; NA: not available; ESAS: Edmonton Symptom Assessment Scale; FACIT: Functional Assessment of Chronic Illness Therapy; FACT: functional assessment of cancer therapy; MM: multiple myeloma; GOG-NTX: Gynecologic Oncology Group-Neurotoxicity; BP, bone pain.

The mean difference in pre- versus post-intervention quality of life (FACT-MM) was +7.3 (95% CI: 0.6–14.0). The mean difference in pre- versus post-intervention fatigue (FACIT-Fatigue subscale—reverse scoring) was +1.7 (95% CI: −1.4 to 4.8). The median difference in pre- versus post-intervention total symptom burden (ESAS global score) was +1 (25th and 75th quartiles: −9 and +11).

## Discussion

Findings from this study indicate that: (1) the program was feasible in terms of recruitment and completion rates, safety, and exercise adherence, (2) participants were satisfied with the program, program staff, and mobile app, and (3) secondary outcome results may indicate promise and warrant further investigation.

### Safety, feasibility, and satisfaction

All feasibility study targets were met. The low attrition rate compares favorably to previously observed rates of 15% to 42%.^12,15,18,54^ Further, adherence to the program was high (82.9%–89.9%). Taken together, these results support the program's feasibility. This suggests that, consistent with the findings of other malignancy groups,^23–26^ an eHealth intervention is a feasible way to deliver an exercise intervention to individuals with MM. This program included three key elements which likely bolstered the program's feasibility: (1) tailoring, (2) virtual supervision/active support, and (3) trained personnel.^20,55,56^

This study is the first to systematically document how programming was tailored to people with MM. Participants required daily/weekly adaptations to keep programming appropriate. Adaptations varied from small adjustments (i.e., alternative exercises) to developing completely new routines. Importantly, myeloma-related pain and a history of fracture/lytic lesions were two primary reasons for program adaptations. These symptoms represent significant barriers to people with MM.^9,57^ In the absence of purposeful adaptations accommodating these symptoms, this program may not be tolerable. Tailoring was facilitated by the functionality of the novel eHealth app, HEAL-Me. HEAL-Me allowed the kinesiologist to easily assign disease-specific template routines to participants, switch out exercises from ≥150 alternatives in seconds, and track when/why adaptations were needed. Given this functionality, the kinesiologist could provide tailored programming with minimal barriers.

Supervision has been proposed to enhance the appropriateness of programming for people with MM.^20^ Indeed, a recent review on eHealth interventions in cancer proposed videoconferencing as a way to deliver accessible, virtually supervised interventions.^22^ In the current study, supervision involved weekly virtually supervised workouts in small group settings and regular participant/specialist check-ins using videoconferencing through HEAL-Me. This allowed for live feedback on exercise form/technique and facilitated discussions that informed exercise adaptations and tailored education/advice.

An appropriately trained and diverse exercise team is likely needed to deliver safe, effective exercise programming in MM. In this study, a kinesiologist with >3 years of experience working with this population delivered the exercise program. The kinesiologist was supported by a physiotherapist with >20 years of experience in cancer rehabilitation who provided program oversight for this and many other programs at our center, a cancer-specific exercise physiologist who completed participant pre-screening, and an oncologist who approved participants for participation. This model aligns with recommendations from the International Bone Metastases Exercise Working Group.^38^ Participants valued this delivery model, identifying staff as knowledgeable, supportive, and comforting. We recommend that future studies in MM carefully consider the need for skilled personnel and interdisciplinary oversight to ensure sufficient safety checks are in place, while keeping program delivery feasible.

No serious life-threatening adverse events or events requiring hospitalization related to the intervention were observed. However, the rate of minor musculoskeletal events was higher compared to a recent exercise trial in MM,^14^ as well as previous studies that reported no adverse events.^11,12,16,18^ It is not clear why the rate of minor musculoskeletal events was higher in the current study compared to previous studies. In-person exercise is sometimes regarded as safer because it allows for both closer monitoring of exercise responses and hands-on assistance with exercise movement or technique.^58^ However, virtually supervised programming has emerged as a feasible and effective alternative for individuals with cancer.^59^ Another possible explanation is that the current study contained a higher proportion of individuals with bone disease, who may be at higher risk of experiencing events, compared to previous studies.^11,12,14,15,17^ In individuals with bone disease, staff must be aware of the potential benefits and risks of exercise, and participants need to make an informed decision on the level of risk that is acceptable to them, given the exercise dose needed to optimize their fitness and function.^38^ Further investigation is warranted to better elucidate the appropriate dosing of exercise in MM in order to deliver both a safe and effective program. In the absence of an established optimal exercise dose in MM, tailoring is likely key in ensuring exercise safety.^20^ Researchers should aim to minimize the likelihood and severity of events by ensuring programming is individually tailored, assisted by active support, and delivered by qualified personnel.^55,56^

### Secondary outcomes

Secondary outcome results may indicate promise and warrant further investigation. The mean change in leg strength (30-s sit-to-stand score) was above the established minimally importance difference (MID) of >2 for older adults and clinical populations,^60,61^ supporting previous findings in MM^12^ and with eHealth interventions in other cancer populations.^27,28^ Prolonged corticosteroid use decreases proximal muscle strength.^36,37^ Lower extremity muscle weakness may increase fall risk,^62^ and therefore, fracture risk^63^ within the MM population. Reducing fall risk through balance exercises and leg/hip muscle strengthening is therefore recommended for future exercise programming for MM.

Although no MID is established for core endurance, the observed mean difference in pre- versus post-intervention plank duration of 41.6 s is promising. Core exercises are often neglected in exercise trials in MM.^11,12,15,17^ Core and back extensor strengthening helps to improve dynamic balance^64^ and posture, reduce axial deformity and back pain,^65,66^ and prevent vertebral fractures in osteoporosis.^67^ The potential benefits of core exercises for improving back pain, posture, and fracture risk, as seen in osteoporosis research, support their inclusion within a myeloma-specific exercise program. However, these exercises should be individually tailored, focus on positioning, and minimize forces on the spine to lower the risk of adverse events and back pain exacerbations,^38^ as observed in this study.

The MM population has a relatively low quality of life and high symptom burden.^68^ In the current study, a pre- versus post-intervention mean difference of +7.3 points was seen in FACT-MM, with the mean change in the emotional subscale achieving the established MID of 2 to 3.^69^ Total symptom burden did not improve in this study, but the program did not exacerbate symptoms. This is important because the control/maintenance of symptoms is a key exercise goal amongst those with advanced cancer.^70^ Symptoms of fatigue, bone pain, and neuropathy were measured in this study. The pre- versus post-intervention change in fatigue was below the MID threshold of 3.^71^ Improved fatigue has been observed in single group studies in MM^12, 72^ but not in randomized control trials.^14,18^ To our knowledge, bone pain and neuropathy have not been outcomes in previous MM exercise trials. There is insufficient evidence on the effects of exercise on neuropathy and pain in cancer.^7^ Further research is warranted to discern the true effects of eHealth programming on overall symptom burden and the burden of specific symptoms/side effects (e.g., fatigue, pain, and neuropathy) in people with MM.

### Limitations

This study is not without limitations. First, without a control group, it is not possible to distinguish between the effect of the treatment and confounders. A single group design was chosen as the focus of this study was feasibility, given the novelty of the virtual delivery. Program effectiveness should be confirmed using a randomized controlled trial. Secondly, gold standard measures of physical function were not used. Valid/reliable virtual assessments were used instead to reach rural/remote participants and follow local COVID-19 public health measures. Thirdly, the sampling method employed in this study introduces referral/selection bias which impacts the generalizability of the results, as participants were likely motivated to exercise at enrollment. Finally, a strength of the program was the use of the HEAL-Me eHealth application to deliver the tailored exercise program. Leveraging the benefits of eHealth could improve access to specialist care for individuals with MM in lower-resource areas, including rural/remote communities.

## Conclusion

The 12-week virtually supported home exercise program was feasible for people with MM but associated with a higher than expected rate of musculoskeletal events, which may be due to the virtual nature of the program or the high proportion of individuals with bone disease included in the study. Programming should be individually tailored, and include supervision, active support, and well-trained personnel in order to manage the possibility of both serious and non-serious adverse events. A randomized controlled trial, with quality of life as a primary outcome, is warranted to determine the effects of the current exercise program. Additional clinically important outcomes including symptom burden, aerobic capacity, leg strength, core strength, and balance should also be explored.

## Supplemental Material

sj-docx-1-dhj-10.1177_20552076221129066 - Supplemental material for Feasibility of a tailored and virtually supported home exercise program for people with multiple myeloma using a novel eHealth application Click here for additional data file. Supplemental material, sj-docx-1-dhj-10.1177_20552076221129066 for Feasibility of a tailored and virtually supported home exercise program for people with multiple myeloma using a novel eHealth application by Graeme M. Purdy, Chris P. Venner, Puneeta Tandon and Margaret L. McNeely in Digital Health
