# Supplementary material for: Feasibility of a tailored and virtually supported home exercise program for people with multiple myeloma using a novel eHealth application
Source: Digit Health. 2022 Oct 11;8:20552076221129066. doi: 10.1177/20552076221129066 (PMC9554139; doi:10.1177/20552076221129066)
Supplement: sj-docx-1-dhj-10.1177_20552076221129066 - Supplemental material for Feasibility of a tailored and virtually supported home exercise program for people with multiple myeloma using a novel eHealth application [file sj-docx-1-dhj-10.1177_20552076221129066.docx]

SUPPLEMENTARY MATERIAL

Table of Contents

[Section 1 – Additional Tailoring/Adaptation Details 2](#_Toc111538108)

[**Table S1. Summary of each unique adaptation where an exercise was switched for an alternative in order to tailor the independent workout to the participant, with counts provided.** 2](#_Toc111538109)

[**Table S2. Summary of each unique adaptation where an original exercise was decreased in difficulty in order to tailor the independent workout to the participant, with counts provided.** 4](#_Toc111538110)

[**Table S3. Summary of each unique adaptation where an original exercise was increased in difficulty in order to tailor the independent workout to the participant, with counts provided.** 5](#_Toc111538111)

[**Table S4. Summary of other adaptations made to independent workouts and the reason/goal behind each of the adaptations, with counts provided.** 5](#_Toc111538112)

[Section 2 – HEAL-Me eHealth App Additional Information 6](#_Toc111538113)

[Section 3 – Virtually Supervised Group Workout Lesson Plans 11](#_Toc111538114)

[**Lesson Plan 1 – Virtually Supervised Group Workout** 11](#_Toc111538115)

[**Lesson Plan 2 – Virtually Supervised Group Workout** 12](#_Toc111538116)

[**Lesson Plan 3 – Virtually Supervised Group Workout** 13](#_Toc111538117)

[**Lesson Plan 4 – Virtually Supervised Group Workout** 14](#_Toc111538118)

[**Lesson Plan 5 – Virtually Supervised Group Workout** 15](#_Toc111538119)

[**Lesson Plan 6 – Virtually Supervised Group Workout** 16](#_Toc111538120)

[**Lesson Plan 7 – Virtually Supervised Group Workout** 17](#_Toc111538121)

[**Lesson Plan 8 – Virtually Supervised Group Workout** 18](#_Toc111538122)

[**Lesson Plan 9 – Virtually Supervised Group Workout** 19](#_Toc111538123)

[**Lesson Plan 10 – Virtually Supervised Group Workout** 20](#_Toc111538124)

[**Lesson Plan 11 – Virtually Supervised Group Workout** 21](#_Toc111538125)

[**Lesson Plan 12 – Virtually Supervised Group Workout** 22](#_Toc111538126)

[Section 4 – Independent Workout Templates 23](#_Toc111538127)

# Section 1 – Additional Tailoring/Adaptation Details

This section contains additional detailed breakdowns of all the adaptations to independent workouts that were completed over the course of the study.

## **Table S1. Summary of each unique adaptation where an exercise was switched for an alternative in order to tailor the independent workout to the participant, with counts provided.**

| Exercise Alternative  Original Exercise | Bicep Curl | Bird Dog | Bow and Arrow Band Pull | Hip Abduction | March | Modified Plank | Modified Side Plank | Mountain Climber | Pec Fly | Plank | Seated Lateral Raise | Seated Leg Extension | Sit to Stand | Split Squat | Squat w/ Knee Raise | Squat w/ Leg Kick | Supine Pec Fly | Trap Setting | Upright Row | Wall Plank |  |
| --- | --- | --- | --- | --- | --- | --- | --- | --- | --- | --- | --- | --- | --- | --- | --- | --- | --- | --- | --- | --- | --- |
| Arnold Press | 1 |  |  |  |  |  |  |  |  |  |  |  |  |  |  |  |  |  |  |  |  |
| Bow and Arrow Band Pull | 1 |  |  |  |  |  |  |  |  |  |  |  |  |  |  |  |  |  |  |  |  |
| Bridge |  | 1 |  |  |  | 1 |  |  |  |  |  |  |  |  |  |  |  |  |  | 1 |  |
| Calf Raise Step |  |  |  |  |  |  |  |  |  |  |  |  |  | 1 |  |  |  |  |  |  |  |
| Clamshell |  |  |  |  |  |  |  |  |  |  |  |  |  | 1 |  |  |  |  |  |  |  |
| Dumbbell Row |  |  | 2 |  |  |  |  |  |  |  |  |  |  |  |  |  |  |  | 1 |  |  |
| Dumbbell Split Squat |  |  |  | 1 |  |  |  |  |  |  |  |  |  |  |  |  |  |  |  |  |  |
| Half Jack |  |  |  |  | 2 |  |  |  |  |  |  |  |  |  |  |  |  |  |  |  |  |
| Hamstring Curl |  |  |  |  |  |  |  |  |  |  |  | 1 | 1 |  |  |  |  |  |  |  |  |
| Modified Deadbug |  |  |  |  |  |  | 1 |  |  | 1 |  |  |  |  |  |  |  |  |  |  |  |
| Modified Pushup |  |  |  |  |  |  |  |  |  |  |  |  |  |  |  |  | 1 |  |  |  |  |
| Sahrmann II |  | 1 |  |  |  |  |  |  |  |  |  |  |  |  |  |  |  |  |  |  |  |
| Seated Lat Pullover |  |  |  |  |  |  |  |  |  |  |  |  |  |  |  |  |  | 1 |  |  |  |
| Seated Shoulder Press |  |  |  |  |  |  |  |  |  |  | 1 |  |  |  |  |  |  |  |  |  |  |
| Side Plank on Wall |  | 1 |  |  |  |  |  |  |  |  |  |  |  |  |  |  |  |  |  |  |  |
| Squat w/ Ball Throw |  |  |  |  |  |  |  |  |  |  |  |  | 1 |  |  |  |  |  |  |  |  |
| Step Toe Taps |  |  |  |  | 1 |  |  |  |  |  |  |  |  |  |  |  |  |  |  |  |  |
| Step Up Kickback |  |  |  |  |  |  |  |  |  |  |  | 1 |  |  |  |  |  |  |  |  |  |
| Step Up Knee Drive |  |  |  |  |  |  |  |  |  |  |  |  | 2 |  |  |  |  |  |  |  |  |
| Step Ups |  |  |  |  |  |  |  | 1 |  |  |  |  |  |  |  |  |  |  |  |  |  |
| Supine Pec Fly |  |  |  |  |  |  |  |  | 1 |  |  |  |  |  |  |  |  |  |  |  |  |
| Upward Plank |  |  |  |  |  | 1 |  |  |  |  |  |  |  |  |  |  |  |  |  |  |  |
| Wall Sit Arm Chop |  |  |  |  |  |  |  |  |  |  |  |  |  |  |  | 1 |  |  |  |  |  |
| Wall Squat |  |  |  | 1 |  |  |  |  |  |  |  |  |  |  | 1 |  |  |  |  |  |  |

## **Table S2. Summary of each unique adaptation where an original exercise was decreased in difficulty in order to tailor the independent workout to the participant, with counts provided.**

| Decreased Difficulty  Original Exercise | Bird Dog | Bow and Arrow Band Pull | Bridging | Calf Raise | Clamshell | March | Modified Deadbug | Modified Plank | One Leg Balance Knee Raise | Sahrmann II | Seated Clamshell | Seated Leg Extension | Seated Pec Fly | Sit to Stand | Split Squat | Step Up Knee Drive | Triceps Kickback |
| --- | --- | --- | --- | --- | --- | --- | --- | --- | --- | --- | --- | --- | --- | --- | --- | --- | --- |
| Bridge w/ Leg Lift | 1 |  |  |  |  |  |  |  |  |  |  |  |  |  |  |  |  |
| Clamshell |  |  |  |  |  |  |  |  |  |  | 2 |  |  |  |  |  |  |
| DB Split Squat |  |  |  |  |  |  |  |  |  |  |  |  |  |  | 1 |  |  |
| Jumping Jack |  |  |  |  |  |  |  |  |  |  |  |  |  |  |  | 2 |  |
| Knee Raise Ball Tap |  |  |  |  |  | 1 |  |  |  |  |  |  |  |  |  |  |  |
| Modified Deadbug |  |  |  |  |  |  |  |  |  | 1 |  |  |  |  |  |  |  |
| Modified Pushup |  |  |  |  |  |  |  |  |  |  |  |  |  |  |  |  | 2 |
| One Leg Balance Shoulder Taps |  |  |  |  |  |  |  |  | 1 |  |  |  |  |  |  |  |  |
| Plie Squat |  |  |  |  |  |  |  |  |  |  |  |  |  | 1 |  |  |  |
| Reverse Lunge |  |  |  | 1 |  |  |  |  |  |  |  |  |  |  |  |  |  |
| Squat w/ Ball Throw |  |  |  |  |  |  |  |  |  |  |  | 2 |  |  |  |  |  |
| Step Ups |  |  |  |  |  |  |  |  |  |  |  |  |  | 1 |  |  |  |
| Supine Pec Fly |  |  |  |  |  |  |  |  |  |  |  |  | 1 |  |  |  |  |
| Upward Plank |  |  | 1 |  |  |  | 3 | 3 |  | 1 |  |  |  |  |  |  |  |
| Wall Sit Arm Chop |  |  |  |  |  |  |  |  |  |  |  |  |  | 1 |  |  |  |
| Weighted Side Lunge |  |  |  |  | 1 |  |  |  |  |  |  |  |  |  |  |  |  |
| YTW |  | 1 |  |  |  |  |  |  |  |  |  |  |  |  |  |  |  |

## **Table S3. Summary of each unique adaptation where an original exercise was increased in difficulty in order to tailor the independent workout to the participant, with counts provided.**

| Increased Difficulty  Original Exercise | Bird Dog | March | Plank | Step Taps | FULL ROUTINE |
| --- | --- | --- | --- | --- | --- |
| Bird Dog (Legs Only) | 3 |  |  |  |  |
| Modified Plank |  |  | 7 |  |  |
| Standing Taps |  |  |  | 2 |  |
| Step Backs |  | 2 |  |  |  |
| FULL ROUTINE |  |  |  |  | 1 |

## **Table S4. Summary of other adaptations made to independent workouts and the reason/goal behind each of the adaptations, with counts provided.**

| Adaptation | Reason/Goal |
| --- | --- |
| Custom Routine | - Extensive bone disease (n=11) - Back pain (n=8) - Balance issues (n=5) |
| Exercise Addition | - Increase volume of core exercises (n=5) - Increase volume of cardio exercises (n=1) |
| Exercise Removal | - Overall volume reduction due to excess load (n=4) - Remove triceps stretch due to limited mobility (n=4) - Remove adductor stretch due to limited mobility (n=1) |
| Order | - Move floor exercises together (n=7) - Move floor exercises away from balance exercise (n=1) - Create sit - stand - sit sequence (n=1) |

# Section 2 – HEAL-Me eHealth App Additional Information

This section contains additional information about the Healthy Eating, Active Living, Mindful Energy (HEAL-Me) eHealth application used in the current study.

The HEAL-Me (Healthy Eating, Active Living, Mindful Energy) app was developed by a multidisciplinary team of patient advisors, behavior change experts, physicians, and allied health specialists (e.g., physical therapists, kinesiologists, dietitians). The app is web-based and can be accessed through any internet connected device (e.g., laptop, desktop computer, iPad or tablet, smartphone, etc.). The app is currently non-commercial and is only accessible participants in research studies that employ the HEAL-Me app. HEAL-Me was specifically designed for use by vulnerable chronic disease populations with a range of digital technology skills. The app uses a simple interface, large buttons, and lay English to address common usability issues prevalent in many eHealth applications. The app is designed to facilitate exercise and nutrition interventions. For the purposes of MY PROGRESS, only the exercise and exercise-related sections were deployed. See Image 1 for a screenshot of the home page of the app. The home page is the landing ground for participants and includes buttons to navigate to each of the key sections of the app, including calendar & live sessions (access to group live workouts & one-on-one check-in sessions), exercise (access to independent home workouts and the independent aerobic exercise tracking portal), achievements (motivational awards marking exercise progress), resources (exercise handouts, safety information, and more), and messages (instant messaging service between participants and the study’s kinesiologist).

Within the calendar & live sessions section, participants are able to sign up for and easily join live group workouts, as well as join their weekly/biweekly check-ins with the study’s kinesiologist (Image 2). In live group workouts, small groups of participants follow along to a live workout lead by the study’s kinesiologist and facilitated by an exercise specialist. The participants’ view shows both exercise trainers side-by-side where they demonstrate two difficulties of the same exercise, so participants with varying abilities can participate in the same class. During live group classes, participants turn their cameras on, so the exercise trainers can view participant posture, technique, and tempo to provide personalized recommendations and encouragements accordingly. For participant privacy reasons, no image is available showing the set-up of the live group workouts. See Image 3 for an example view of the set-up the exercise trainers had when leading lead these classes. Set-up included a large TV to easily view participants, a wide-angle 1080-pixel webcam, a tripod to mount the webcam, an exercise mat, bands, free weights, step/box, free wall space, open space, and proper lighting.

Within the exercise section, participants can track their independent aerobic exercise, shortcut back to the live group workout section of the app or enter the independent home workout section (Image 4). See Image 5 to view the format of the independent home workouts from the participant’s view. Exercise videos automatically play sequentially in a circuit-style format (see further details below about the resistance exercise prescription). Participants can maximize/minimize the video window, pause/play, or scroll down to view a written description of the circuit and upcoming exercises. This description includes the exercise names still images of each exercise, a list of required equipment, and the level of difficulty of each exercise. All exercise videos include an exercise specialist on screen who explains the exercise and provides cues/tips and a patient with lived experience with chronic disease who acts as an exercise demonstrator.

To track/log live group workouts, independent home workouts, and independent aerobic exercise, participants use a tracking feature within the HEAL-Me app. Following workouts, participants are prompted to record their rating of perceived exertion and log their workout (Image 6). Participants are also able to log their independent aerobic exercise using an identical screen to the one shown in Image 6.

Image 1. Screenshot of the home page of the HEAL-Me App.


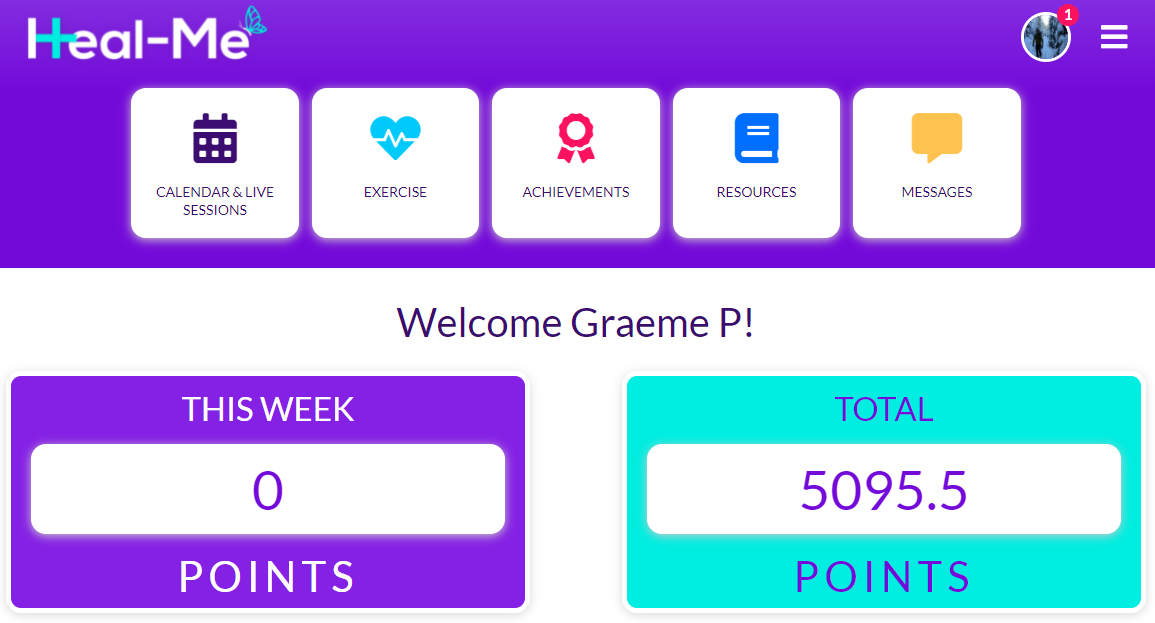


Image 2. Screenshot of the main view in the calendar & live session section of the app.


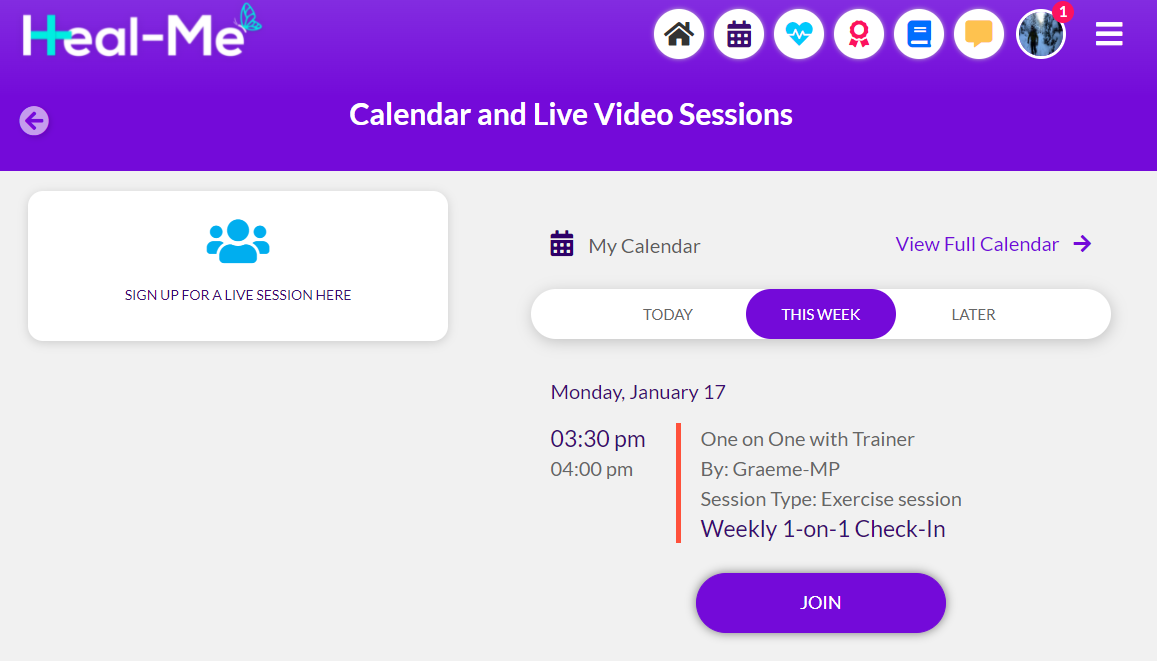


Image 3. Example of the study kinesiologist’s set-up when leading the live group workouts, from the perspective of the TV monitor.


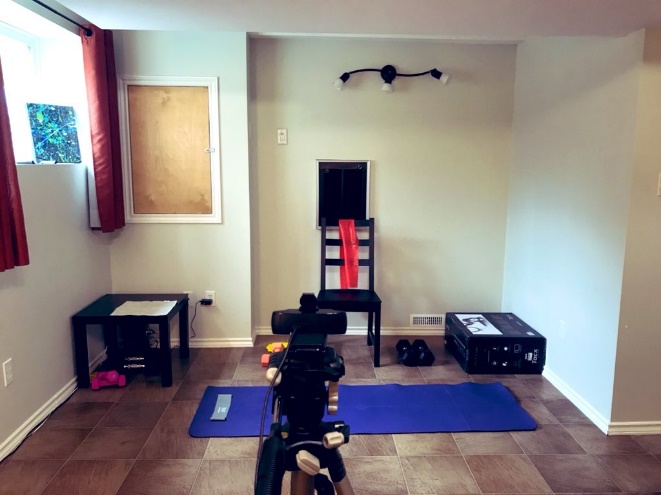


Image 4. Screenshot of the main page within the exercise section of the app.


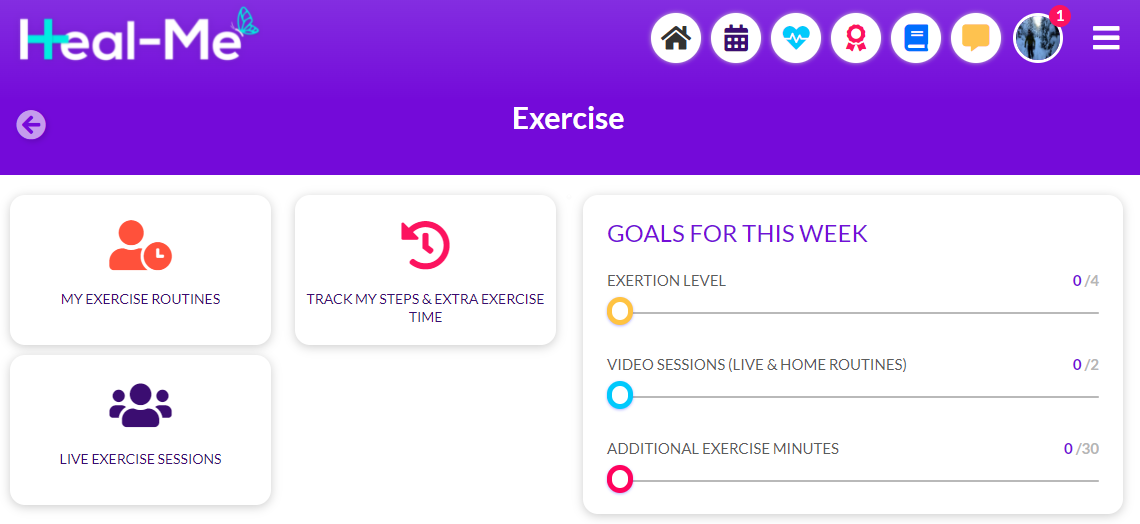


Image 5. Screenshot of the participant view for the independent home workouts.


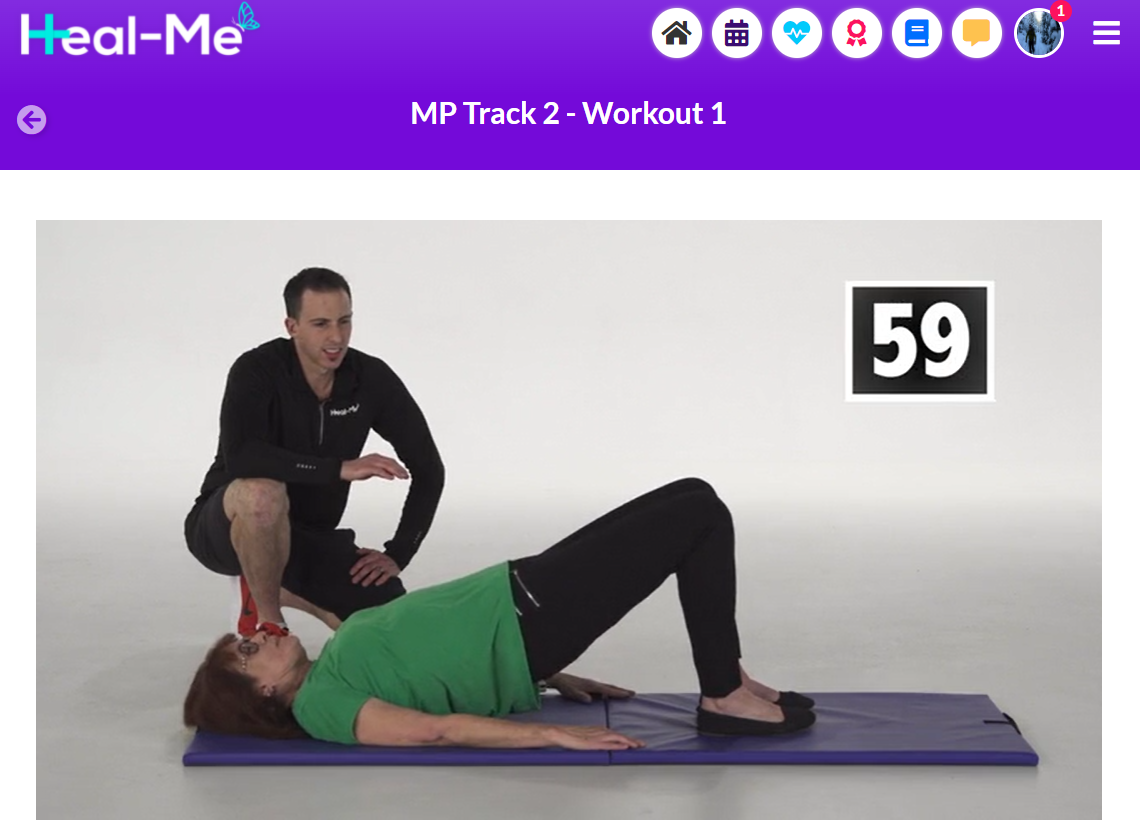


Image 6. Screenshot of the workout session and rating of perceived exertion tracking page.


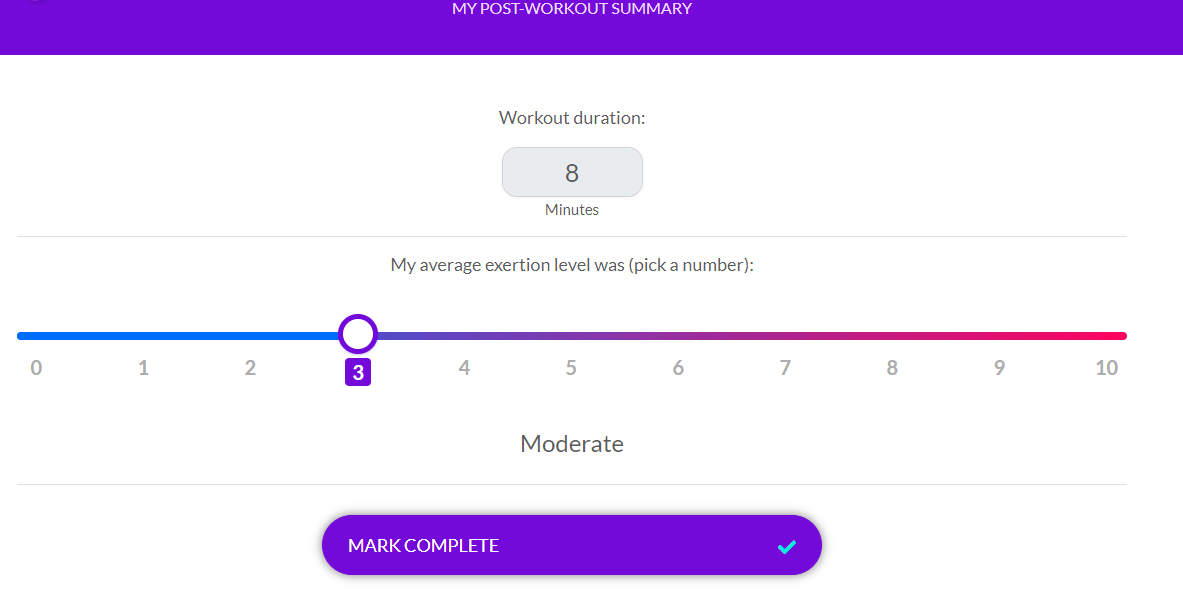


# Section 3 – Virtually Supervised Group Workout Lesson Plans

This section contains the original lesson plans for the virtually supervised group workouts delivered in the study.

## **Lesson Plan 1 – Virtually Supervised Group Workout**

WARM UP

2 rounds of the following exercises

- Chest Opens
- Calf Raises
- High Knees
- High Knee Pulldowns
- Butt Kicks
- High Row Butt Kicks
- Shallow Squat

CIRCUIT

2 rounds of 8 exercises, 60 seconds work, 30 seconds rest

| Exercise | Type | Level 1 | Level 2 |
| --- | --- | --- | --- |
| 1 | Cardio | No Weights | Kickbox Punch Knee Drive (R, L, R, Left Knee Drive) |
| 2 | Lower Body | Standing Leg Abduction (w/ or w/o band) | Clamshell (w/ or w/o band) |
| 3 | Upper Body | Triceps Kickback (Band or DB) | Supine Triceps Extension |
| 4 | Core | High Plank (Chair) | High Plank (Floor) |
| 5 | Cardio | High Knee March | Step Ups |
| 6 | Upper Body | --- | Lat Pulldown |
| 7 | Lower Body | Squat or Sit-to-Stand (DB) | Squat w/ Knee Raise (DB) |
| 8 | Balance | Drinking Bird (Shallow) | Drinking Bird (Deep) |

CORE

2 rounds – 60 seconds work, 30 seconds rest

| Exercise # | Type | Exercise |
| --- | --- | --- |
| 1 | Core | Baby Cobra 🡪 Superman |
| 2 | Core | Wall Plank 🡪 Knee Plank 🡪 Full Plank |

## **Lesson Plan 2 – Virtually Supervised Group Workout**

WARM UP

2 rounds of the following exercises

- March
- March + Arm Swings
- Butt Kicks
- Butt Kicks + High Row
- Shallow Squat
- Shallow Squat + Shoulder Press

CIRCUIT

2 rounds of 8 exercises, 60 seconds work, 30 seconds rest

| Exercise | Type | Level 1 | Level 2 |
| --- | --- | --- | --- |
| 1 | Cardio | No Weights | Kickbox Punch Knee Drive (R, L, R, Left Knee Drive) |
| 2 | Lower Body | Standing Leg Abduction (w/ or w/o band) | Clamshell (w/ or w/o band) |
| 3 | Upper Body | Chest Fly (Seated) | Prone Chest Fly (DB) |
| 4 | Core | High Plank (Chair) | High Plank (Floor) |
| 5 | Cardio | Half Jack (Uni or Bilateral) | Half Jack on Step (Uni or Bilateral) |
| 6 | Upper Body | --- | Lat Pulldown |
| 7 | Lower Body | Sit to Stand + Bicep Curl (or Squat) | Wall Sit + Bicep Curl |
| 8 | Balance | Heel-Toe Walk | Line Walk + High Knee |

BALANCE

2 rounds – Complete exercise with right leg raised then repeat with leg left raised.

| Exercise # | Type | Exercise |
| --- | --- | --- |
| 1 | BALANCE | 1. Right foot into hamstring curl, arms go back and squeeze (5-10s) 2. Knee up, hand into bicep curl and squeeze (5-10s)   Repeat for 4x |
| 2 | BALANCE | Repeat on other leg |

## **Lesson Plan 3 – Virtually Supervised Group Workout**

WARM UP

2 rounds of the following exercises

- March
- March + Arm Swings
- Butt Kicks
- Butt Kicks + High Row
- Shallow Squat
- Shallow Squat + Shoulder Press

CIRCUIT

2 rounds of 8 exercises, 60 seconds work, 30 seconds rest

| Exercise | Type | Level 1 | Level 2 |
| --- | --- | --- | --- |
| 1 | Cardio | Step Knee Drive | Step Up Knee Drive |
| 2 | Lower Body | Split Stance Lunge | Reverse Lunge (+/- Weights) |
| 3 | Upper Body | Chest Fly (Seated) | Prone Chest Fly (DB) |
| 4 | Core | On Chair (or Higher Surface) | Core Mountain Climber |
| 5 | Cardio | Half Jack (Uni or Bilateral) | Half Jack on Step (Uni or Bilateral) |
| 6 | Upper Body | Front Raise (Seated if w/ Band) | Arnold Press |
| 7 | Lower Body | Sit to Stand + Bicep Curl (or Squat) | Wall Sit + Bicep Curl |
| 8 | Balance | Heel-Toe Walk | Line Walk + High Knee |

CORE

2 rounds – 60 seconds work, 30 seconds rest

| Exercise # | Type | Exercise |
| --- | --- | --- |
| 1 | CORE | Side Plank on Wall 🡪 Side-Lying Hip Raises 🡪 Side Plank |
| 2 | CORE | Baby Cobra 🡪 Superman |

## **Lesson Plan 4 – Virtually Supervised Group Workout**

WARM UP

2 rounds of the following exercises

- March
- March w/ Shrugs
- Step Touch
- Step Touch w/ Jab
- Out-Out, In-In
- Out-Out, In-In w/ Shoulder Raise
- Butt Kick +/- Squat
- Butt Kick (+/- Squat) w/ High Row

CIRCUIT

2 rounds of 8 exercises, 60 seconds work, 30 seconds rest

| Exercise | Type | Level 1 | Level 2 |
| --- | --- | --- | --- |
| 1 | Cardio | Step Knee Drive | Step Up Knee Drive |
| 2 | Lower Body | Bow/Arrow Band Pull (Seated) | Bow/Arrow Band Pull |
| 3 | Upper Body | Donkey Kick (or Standing) | Single Leg Bridge |
| 4 | Core | On Chair (or Higher Surface) | Core Mountain Climber |
| 5 | Cardio | Pulldown Knee Drive (w/o Kick) | Pulldown Knee Drive (w/ Kick) |
| 6 | Upper Body | Front Raise (Seated if w/ Band) | Arnold Press |
| 7 | Lower Body | Inline Lunge | Reverse Lunge |
| 8 | Balance | 1 Foot Hip Taps (or Static) | 1 Foot Shoulder Taps |

BALANCE

Complete once through. Can repeat 2^nd^ time if time permits.

| Exercise # | Type | Exercise |
| --- | --- | --- |
| 1 | Balance | BB Right Hand, Left Leg Up. Toe Point (Squeeze) 🡪 Knee Raise (Squeeze) 🡪 Side Point (Squeeze) 🡪 Behind (Squeeze) 🡪 Hamstring Curl (Squeeze) |
| 2 | Balance | Repeat on Other Leg |

## **Lesson Plan 5 – Virtually Supervised Group Workout**

WARM UP

2 rounds of the following exercises

- March
- March w/ Shrugs
- Step Touch
- Step Touch w/ Jab
- Out-Out, In-In
- Out-Out, In-In w/ Shoulder Raise
- Butt Kick +/- Squat
- Butt Kick (+/- Squat) w/ High Row

CIRCUIT

2 rounds of 8 exercises, 60 seconds work, 30 seconds rest

| Exercise | Type | Level 1 | Level 2 |
| --- | --- | --- | --- |
| 1 | Cardio | Half Jack | Half Jack on Step |
| 2 | Lower Body | Bow/Arrow Band Pull (Seated) | Bow/Arrow Band Pull |
| 3 | Upper Body | Donkey Kick (or Standing) | Single Leg Bridge |
| 4 | Core | Plank (knees or high surface) | Plank |
| 5 | Cardio | Pulldown Knee Drive (w/o Kick) | Pulldown Knee Drive (w/ Kick) |
| 6 | Upper Body | --- | Trap Setting |
| 7 | Lower Body | Deadlift Hinge (Band or DB) | Deadlift Hinge (Band or DB) |
| 8 | Balance | 1 Foot Hip Taps (or Static) | 1 Foot Shoulder Taps |

CORE

2 rounds – 60 seconds work, 30 seconds rest

| Exercise # | Type | Exercise |
| --- | --- | --- |
| 1 | Core | Wall Plank 🡪 Knee Plank 🡪 Full Plank |
| 2 | Core | Legs-Only Bird Dog 🡪 Full Bird Dog |

## **Lesson Plan 6 – Virtually Supervised Group Workout**

WARM UP

2 rounds of the following exercises

- Leg Swings
- Snow Angels
- Sideways Toe Taps
- Half Jack
- High Knee
- Pull Down High Knee
- Out-Out, In-In
- Out-Out, In-In w/ Shoulder Raise

CIRCUIT

2 rounds of 8 exercises, 60 seconds work, 30 seconds rest

| Exercise | Type | Level 1 | Level 2 |
| --- | --- | --- | --- |
| 1 | Cardio | Half Jack | Half Jack on Step |
| 2 | Lower Body | --- | Deadlift Hinge (Band or DB) |
| 3 | Upper Body | Supine Chest Press | Seated Chest Press w/ Band |
| 4 | Core | Bird Dog (Legs Only) | Bird Dog |
| 5 | Cardio | Sit to Stand | Weighted Sit to Stand |
| 6 | Upper Body | --- | Trap Setting with Towel/Band/Shirt |
| 7 | Lower Body | Reverse Lunge | Reverse Lunge w/ Knee Drive |
| 8 | Balance | 1 Foot Balance | 1 Foot Alphabet |

BALANCE

2 rounds – Complete exercise 1 and 2 with right leg raised then repeat both with leg left raised.

| Exercise # | Type | Exercise |
| --- | --- | --- |
| 1 | Balance | Ball/Towel/Shirt in right hand. Raise the right foot off the ground.  With the right knee up,  - Raise the arm directly in front, squeeze for 5 seconds  - Raise the arm overhead, squeeze for 5 seconds  - Raise the arm to the side, squeeze for 5 seconds  - Raise the arm behind, squeeze for 5 seconds |
| 2 | Balance | Ball/Towel/Shirt in right hand. Raise the right foot off the ground.  With the right knee bent backward in a hamstring curl,  - Raise the arm directly in front, squeeze for 5 seconds  - Raise the arm overhead, squeeze for 5 seconds  - Raise the arm to the side, squeeze for 5 seconds  - Raise the arm behind, squeeze for 5 seconds |

## **Lesson Plan 7 – Virtually Supervised Group Workout**

WARM UP

2 rounds of the following exercises

- Snow Angels
- Lateral Line Stepovers
- Forward-Backward Line Stepovers
- Forward-Backward Line Stepovers w/ Bicep Curl
- Sideways Toe Taps
- Half Jack w/ Forward Arm Reach
- High Knee
- Pull Down High Knee

CIRCUIT

2 rounds of 8 exercises, 60 seconds work, 30 seconds rest

| Exercise | Type | Level 1 | Level 2 |
| --- | --- | --- | --- |
| 1 | Cardio | Mountain Climber Against High Surface | Mountain Climber Against Low Surface |
| 2 | Lower Body | Seated Chest Press w/ Band | Supine Chest Press |
| 3 | Upper Body | Side-Lie Hip Abduction | Side-Lie Hip Abduction w/ Band |
| 4 | Core | Bird Dog | Bird Dog (Legs Only) |
| 5 | Cardio | Weighted Sit to Stand | Sit to Stand |
| 6 | Upper Body | Seated Reverse Fly | Reverse Fly |
| 7 | Lower Body | Air Squat | Wall Sit |
| 8 | Balance | 1 Foot Balance | 1 Foot Alphabet |

CORE

2 rounds – 60 seconds work, 30 seconds rest

| Exercise # | Type | Exercise |
| --- | --- | --- |
| 1 | Core | Legs-Only Bird Dog 🡪 Glute Bridge 🡪 Single Leg Glute Bridge |
| 2 | Core | Wall Plank 🡪 Knee Plank 🡪 Full Plank |

## **Lesson Plan 8 – Virtually Supervised Group Workout**

WARM UP

2 rounds of the following exercises

- Snow Angels
- Lateral Line Stepovers
- Forward-Backward Line Stepovers
- Forward-Backward Line Stepovers w/ Bicep Curl
- Sideways Toe Taps
- Half Jack w/ Forward Arm Reach
- High Knee
- Pull Down High Knee

CIRCUIT

2 rounds of 8 exercises, 60 seconds work, 30 seconds rest

| Exercise | Type | Level 1 | Level 2 |
| --- | --- | --- | --- |
| 1 | Cardio | Mountain Climber Against High Surface | Mountain Climber Against Low Surface |
| 2 | Lower Body | Front Raise | Arnold Press |
| 3 | Upper Body | Side-Lie Hip Abduction | Side-Lie Hip Abduction w/ Band |
| 4 | Core | Modified Deadbug | Deadbug |
| 5 | Cardio | Kickbox Punch Knee Drive w/o Weights | Kickbox Punch Knee Drive w/ Weights |
| 6 | Upper Body | Seated Reverse Fly | Reverse Fly |
| 7 | Lower Body | Air Squat | Wall Sit |
| 8 | Balance | Line Walk | Line Walk w/ High Knee |

BALANCE

2 rounds – Complete exercise 1 and 2 with right leg raised then repeat both with leg left raised. Repeat each 5x.

| Exercise # | Type | Exercise |
| --- | --- | --- |
| 1 | Balance | Right hand in bicep curl, right food in hamstring curl. Squeeze (5-10s). |
| 2 | Balance | Right hand backward, right foot in knee raise. Squeeze (5-10s). |

## **Lesson Plan 9 – Virtually Supervised Group Workout**

WARM UP

2 rounds of the following exercises

- Snow Angels
- Lateral Line Stepovers
- Forward-Backward Line Stepovers
- Forward-Backward Line Stepovers w/ Bicep Curl
- Sideways Toe Taps
- Half Jack w/ Forward Arm Reach
- High Knee
- Pull Down High Knee

CIRCUIT

2 rounds of 8 exercises, 60 seconds work, 30 seconds rest

| Exercise | Type | Level 1 | Level 2 |
| --- | --- | --- | --- |
| 1 | Cardio | High Knee Tap | High Knee Tap w/ Resistance |
| 2 | Lower Body | Shallow Reverse Lunge | Reverse Lunge w/ Resistance |
| 3 | Upper Body | Standing YTW | Prone YTW |
| 4 | Core | Easier Deadbug | Deadbug |
| 5 | Cardio | Kickbox Punch Knee Drive w/o Weights | Kickbox Punch Knee Drive w/ Weights |
| 6 | Upper Body | Front Raise | Arnold Press |
| 7 | Lower Body | Sit-to-Stand Press | Squat Press |
| 8 | Balance | Line Walk | Line Walk w/ High Knee |

CORE

2 rounds – 60 seconds work, 30 seconds rest

| Exercise # | Type | Exercise |
| --- | --- | --- |
| 1 | Core | Wall Plank 🡪 Knee Plank 🡪 Full Plank |
| 2 | Core | Wall Side Plank w/ Leg Raise 🡪 Knee Side Plank w/ Leg Raise |

## **Lesson Plan 10 – Virtually Supervised Group Workout**

WARM UP

2 rounds of the following exercises

- Y-T-W
- Standing Calf Raises
- Step Touch
- Step Touch w/ Arm Reach
- Out-Out In-In
- Out-Out In-In w/ Lat Raises
- Mini Squat
- Mini Squat w/ Jab

CIRCUIT

2 rounds of 8 exercises, 60 seconds work, 30 seconds rest

| Exercise # | Type | Level 1 | Level 2 |
| --- | --- | --- | --- |
| 1 | Cardio | High Knee Tap | High Knee Tap w/ Resistance |
| 2 | Lower Body | Shallow Reverse Lunge | Reverse Lunge w/ Resistance |
| 3 | Upper Body | Standing YTW | Prone YTW |
| 4 | Core | Static Bridge | Marching Bridge |
| 5 | Cardio | Kickbox Triple Step Small Knee Drive | Kickbox Triple Step Knee Drive |
| 6 | Upper Body | Bicep Curl (Seated) | Bicep Curl |
| 7 | Lower Body | Sit-to-Stand Press | Squat Press |
| 8 | Balance | 3 Way Foot Reach | 3 Way Foot Reach (Arms Out) |

BALANCE

3 rounds – Complete exercise with right leg raised then repeat with leg left raised.

| Exercise # | Type | Exercise |
| --- | --- | --- |
| 1 | Balance | Drinking Bird – Standing on right foot, towel in left hand. Hinge forward at hip. Raise arm to front raise, squeeze. lower arm beside body, Squeeze. Raise arm to reverse raise, squeeze. |
| 2 | Balance | Repeat on Other Leg. |

## **Lesson Plan 11 – Virtually Supervised Group Workout**

WARM UP

2 rounds of the following exercises

- Y-T-W
- Standing Calf Raises
- Step Touch
- Step Touch w/ Arm Reach
- Out-Out In-In
- Out-Out In-In w/ Lat Raises
- Mini Squat
- Mini Squat w/ Jab

CIRCUIT

2 rounds of 8 exercises, 60 seconds work, 30 seconds rest

| Exercise # | Type | Level 1 | Level 2 |
| --- | --- | --- | --- |
| 1 | Cardio | Low-Impact Burpee (high surface) | Low Impact Burpee (low surface) |
| 2 | Lower Body | Stationary Side Lunge | Side Lunge w/ Leg Raise |
| 3 | Upper Body | Seated Band Row | DB Row |
| 4 | Core | Static Bridge | Marching Bridge |
| 5 | Cardio | Kickbox Triple Step Small Knee Drive | Kickbox Triple Step Knee Drive |
| 6 | Upper Body | Bicep Curl (Seated) | Bicep Curl |
| 7 | Lower Body | Sit to Stand (w/ or w/o weights) | Wall Sit |
| 8 | Balance | 3 Way Foot Reach | 3 Way Foot Reach (Arms Out) |

CORE

2 rounds – 60 seconds work, 30 seconds rest

| Exercise # | Type | Exercise |
| --- | --- | --- |
| 1 | Core | Side Lying Leg Raise (1 or Both Legs) |
| 2 | Core | Bird Dog with or without arms |

## **Lesson Plan 12 – Virtually Supervised Group Workout**

WARM UP

2 rounds of the following exercises

- Snow Angels
- Side Lunge Overhead Reach
- Step Touch
- Step Touch w/ Downward Reach
- Forward-Backward Line Stepovers
- FB Stepovers w/ Bent Elbow Shoulder Fly
- Half Jack
- Squat Press (or Sit-to-Stand Press)

CIRCUIT

2 rounds of 8 exercises, 60 seconds work, 30 seconds rest

| Exercise # | Type | Level 1 | Level 2 |
| --- | --- | --- | --- |
| 1 | Cardio | Low-Impact Burpee (high surface) | Low Impact Burpee (low surface) |
| 2 | Lower Body | Stationary Side Lunge | Side Lunge w/ Leg Raise |
| 3 | Upper Body | Bent-Over Triceps Extension | Supine Triceps Extension |
| 4 | Core | High Plank (Chair) | High Plank (Floor) |
| 5 | Cardio | High Knee March | Step Ups |
| 6 | Upper Body | Seated Band Row | DB Row |
| 7 | Lower Body | Sit to Stand (w/ or w/o weights) | Wall Sit |
| 8 | Balance | Same, but less deep | Drinking Bird/Airplane Pose |

BALANCE

2 rounds – Complete exercise with right leg raised then repeat with leg left raised.

| Exercise # | Type | Exercise |
| --- | --- | --- |
| 1 | Core | Wall Plank 🡪 Knee Plank 🡪 Full Plank |
| 2 | Core | Seated Deadbug 🡪 Modified Deadbug 🡪 Deadbug |

# Section 4 – Independent Workout Templates

This section contains a summary of the original independent workout templates using in the study. Adaptations to these templates were made as needed to ensure the program was tailored to each participant’s abilities, goals, and preferences.

| Workout | Warm-Up | Cardio | Upper Body | Lower Body | Core | Balance |
| --- | --- | --- | --- | --- | --- | --- |
| TRACK 1 | | | | | | |
| 1 (Starting Point #1) | Video #1 | - Seated March - Seated Half Jack | - Seated Bicep Curl - Seated Lateral Raise | - Calf Raise - Seated Clamshell | - Wall Plank | - Tandem Stance |
| 2 | Video #1 | - Seated March - Standing Toe Taps | - Seated Bicep Curl - Seated Pec Fly | - Calf Raise - Sit to Stand | - Wall Plank | - Tandem Stance |
| 3 | Video #2 | - Step Backs - Standing Toe Taps | - Seated Reverse Fly - Seated Pec Fly | - Step Up Kickback - Sit to Stand | - Bird Dog (Legs Only) | - Single Leg Balance |
| 4 (Starting Point #2) | Video #2 | - Step Backs - Forward-Back Stepovers | - Seated Reverse Fly - Wall Push Up | - Step Up Kickback - Hamstring Curl | - Bird Dog (Legs Only) | - Single Leg Balance |
| 5 | Video #3 | - Standing March - Forward-Back Stepovers | - Seated Row - Wall Push Up | - Bodyweight Squat - Hamstring Curl | - Side Plank on Wall | - Tandem Stance (Narrow) |
| 6 | Video #3 | - Standing March - Half Jack | - Seated Row - Seated Shoulder Press | - Bodyweight Squat - Clamshell | - Side Plank on Wall | - Tandem Stance (Narrow) |
| 7 (Starting Point #3) | Video #4 | - Step Toe Taps - Half Jack | - Standing Bicep Curl - Seated Shoulder Press | - Plie Squat - Clamshell | - Bridging | - Tree Pose |
| 8 | Video #4 | - Step Toe Taps - Modified Mountain Climber | - Standing Bicep Curl - Standing Lateral Raise | - Plie Squat - Calf Raise on Step | - Bridging | - Tree Pose |
| 9 | Video #5 | - Lateral Line Stepovers - Modified Mountain Climber | - Standing Reverse Fly - Standing Lateral Raise | - Wall Squat - Calf Raise from Step | - Knee Plank | - Single Leg Balance w/ Knee Raise |
| 10 | Video #5 | - Lateral Line Stepovers - Step Ups | - Standing Reverse Fly - Triceps Kickback | - Wall Squat - Squat w/ Knee Raise | - Knee Plank | - Single Leg Balance w/ Knee Raise |
| 11 | Video #6 | - Knee Raise w/ Ball Tap - Step Ups | - Trap Setting - Triceps Kickback | - Wall Sit Arm Chop - Squat w/ Knee Raise | - Knee Side Plank | - One Foot Shoulder Taps |
| 12 | Video #6 | - Knee Raise w/ Ball Tap - Half Jack | - Trap Setting - Bow and Arrow Band Pull | - Wall Sit Arm Chop - Split Squat | - Knee Side Plank | - One Foot Shoulder Taps |
| TRACK 2  Continuation of track 1. Begins with slight demotion of exercise difficulty, but focus on ↑ pace on cardio, ↑ resistance on upper/lower body, ↓ use of support on balance, ↑ rest on core. | | | | | | |
| 13 (Starting Point #3) | Video #5 | - Standing March - Step Toe Taps | - Standing Bicep Curl - Trap Setting | - Sit to Stand - Clamshell | - Bridging w/ Band | - Single Leg Balance |
| 14 | Video #5 | - Standing March - Half Jack | - Standing Bicep Curl - Standing Lateral Raise | - Sit to Stand - Plie Squat | - Bridging w/ Band | - Single Leg Balance |
| 15 | Video #6 | - Modified Mountain Climber - Half Jack | - Bow and Arrow Band Pull - Standing Lateral Raise | - Squat w/ Ball Throw - Plie Squat | - Bird Dog | - Tree Pose |
| 16 (Starting Point #4) | Video #6 | - Modified Mountain Climber - Forward-Back Stepovers | - Bow and Arrow Band Pull - Triceps Kickback | - Squat w/ Ball Throw - Calf Raise from Step | - Bird Dog | - Tree Pose |
| 17 | Video #7 | - Step Ups - Forward-Back Stepovers | - Standing Reverse Fly - Triceps Kickback | - Squat w/ Knee Raise - Calf Raise from Step | - Knee/Toe Plank | - Single Leg Balance w/ Knee Raise |
| 18 | Video #7 | - Step Ups - Lateral Line Stepovers | - Standing Reverse Fly - Supine Pec Fly | - Squat w/ Knee Raise - Clamshell | - Knee/Toe Plank | - Single Leg Balance w/ Knee Raise |
| 19 | Video #8 | - Knee Raise w/ Ball Tap - Lateral Line Stepovers | - Dumbbell Row - Supine Pec Fly | - Squat w/ Leg Kick - Clamshell | - Modified Deadbug | - One Foot Shoulder Taps |
| 20 | Video #8 | - Knee Raise w/ Ball Tap - Step Ups w/ Knee Drive | - Dumbbell Row - Standing Arnold Press | - Squat w/ Leg Kick - Weighted Side Lunge | - Modified Deadbug | - One Foot Shoulder Taps |
| 21 | Video #9 | - Half Jack - Advanced Mountain Climber | - Reverse Fly w/ ¼ squat - Standing Arnold Press | - Wall Sit - Weighted Side Lunge | - Bridge w/ Leg Lift | - Single Leg Alphabet |
| 22 | Video #9 | - Half Jack - Advanced Mountain Climber | - Reverse Fly w/ ¼ squat - Modified Push Up Or YTW | - Wall Sit - Dumbbell Split Squat | - Bridge w/ Leg Lift | - Single Leg Alphabet |
| 23 | Video #10 | - Step Down Reach - Advanced Mountain Climber | - Upright Row - Modified Push Up Or YTW | - Wall Sit Arm Chop - Dumbbell Split Squat | - Upward Plank | - 3-Way Foot Reach |
| 24 | Video #10 | - Step Down Reach - Jumping Jack | - Upright Row - Arnold Press w/ ¼ Squat | - Wall Sit Arm Chop - Plie Squat w/ Resistance | - Upward Plank | - 3-Way Foot Reach |

NOTES:

- Warm-Ups: participants had the option to complete the follow-along warm-up video or complete an aerobic exercise of equivalent length of their choosing
- Secondary Circuit: on an alternating basis, each workout was followed by a core circuit, balance circuit, or additional stretching. Core circuits and balance circuits were copies of the circuit used in the corresponding week’s virtually supervised group workout lesson plan. Additional stretching added a second repetition of stretching included in each workout’s cooldown
- Cool-Down: each workout was finished with a stretching sequence of the key muscle groups (i.e., quadriceps, hamstrings, calves, glutes, chest, triceps, back).
- No participants started at starting point #4, so no workout templates beyond #24 are shown.
